# Bridging sensory and language theories of dyslexia: Toward a multifactorial model

**DOI:** 10.1111/desc.13039

**Published:** 2020-10-19

**Authors:** Gabrielle O’Brien, Jason D. Yeatman

**Affiliations:** ^1^ Institute for Learning & Brain Sciences University of Washington Seattle WA USA; ^2^ Department of Speech and Hearing Sciences University of Washington Seattle WA USA; ^3^ Graduate School of Education Stanford University Stanford CA USA; ^4^ Division of Developmental‐Behavioral Pediatrics Stanford University School of Medicine Stanford CA USA

**Keywords:** deficit, dyslexia, learning, phonological, psychophysics, reading, visual

## Abstract

Competing theories of dyslexia posit that reading difficulties arise from impaired visual, auditory, phonological, or statistical learning mechanisms. Importantly, many theories posit that dyslexia reflects a cascade of impairments emanating from a single “core deficit”. Here we report two studies evaluating core deficit and multifactorial models. In Study 1, we use publicly available data from the Healthy Brain Network to test the accuracy of phonological processing measures for predicting dyslexia diagnosis and find that over 30% of cases are misclassified (sensitivity = 66.7%; specificity = 68.2%). In Study 2, we collect a battery of psychophysical measures of visual motion processing and standardized measures of phonological processing in 106 school‐aged children to investigate whether dyslexia is best conceptualized under a core‐deficit model, or as a disorder with heterogenous origins. Specifically, by capitalizing on the drift diffusion model to analyze performance on a visual motion discrimination experiment, we show that deficits in visual motion processing, perceptual decision‐making, and phonological processing manifest largely independently. Based on statistical models of how variance in reading skill is parceled across measures of visual processing, phonological processing, and decision‐making, our results challenge the notion that a unifying deficit characterizes dyslexia. Instead, these findings indicate a model where reading skill is explained by several distinct, additive predictors, or risk factors, of reading (dis)ability.

## Graphical abstract

Using predictors from a visual motion processing experiment and linguistic measures, we show that a single‐mechanism model of reading disability cannot account for the range of linguistic and sensory processing outcomes observed in children. We propose an additive risk factor model where different aspects of sensory, cognitive and language function each contribute independently to reading development.

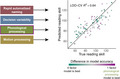

**Research Highlights**

- New evidence that a single‐mechanism model of dyslexia cannot account for the range of linguistic and sensory processing outcomes in children.
- Contrary to many previous hypotheses, our data suggest that predictors from visual motion processing experiments can influence reading skill independently of phonological processing.
- We propose an additive risk factor model where different aspects of sensory, cognitive, and language function each contribute independently to reading development.

## 1 BACKGROUND

Recently, there has been growing adoption of the view that dyslexia, a reading disability, is probabilistic in nature; children with a family history of dyslexia are considered “at‐risk”, and compensatory skills such as strong oral language or executive functions may be “protective factors” (Haft et al., 2016; Hulme et al., 2015; Muter & Snowling, 2009; Pennington, 2006). In this multifactorial framework, most cases of dyslexia cannot be explained by a single cognitive deficit. Despite this heterogeneity, it is broadly accepted that phonological awareness (PA) and rapid automatized naming (RAN) are two of the strongest—if imperfect—predictors of reading development (Pennington et al., 2012; Wolf & Bowers, 2000).

In parallel, there is a broad literature characterizing dyslexia as the consequence of a fundamental deficit that supersedes phonological processing. There are many reports indicating that people with dyslexia perform poorly in experiments targeting various aspects of visual (Stuart et al., 2006; Talcott et al., 2002) and auditory processing (Hämäläinen et al., 2013; Noordenbos & Serniclaes, 2015), as well domain general mechanisms such as processing speed and statistical learning (Gabay et al., 2015; Vandermosten et al., 2018). These findings have spurred competing theories that explain dyslexia as the consequence of cascading effects from a fundamental deficit in the neural systems that process sensory information (either visual, auditory, or both; Goswami, 2015), or the ability to make optimal use of sensory information (e.g., Ahissar, 2007; Ramus & Ahissar, 2012). Sensory systems are organized in a hierarchy and the information that is encoded by the eyes and ears is processed in a sequence of stages in the brain. Generally, these “cascading deficit” theories contend that deficits in one of the early stages of sensory processing disrupt the development of phonological processing and, by this mechanism, disrupt reading development.

Notably, these two branches of research remain largely distinct; while multifactorial models of reading disability are increasingly accepted among researchers studying high‐level cognitive and linguistic functions, these models largely ignore lower level deficits in sensory processing. In the sensory‐processing literature, on the other hand, cascading deficit models continue to dominate and appeals to a “core mechanism” of dyslexia are still commonplace. Indeed, a PubMed search for the phrase “core deficit of dyslexia” turns up 118 results from 1986 to the present. Presently, hypotheses positing a core deficit with cascading effects are the focus of many neuroscientific and psychophysical studies of reading disability (Casini et al., 2018; Colling et al., 2017; Frey, François, Chobert, Besson, et al., 2019; Frey, François, Chobert, Velay, et al., 2019; Gori et al., 2016; Krause, 2015; Lieder et al., 2019; Nicolson & Fawcett, 2018; Vidyasagar, 2019).

A core deficit model is inherently at odds with a multifactorial model; to accept both models implies that a deficit is not really “core” in the majority of individuals with dyslexia. Reconciling the many disparate theories of reading disability remains a formidable challenge. To further compound the difficulty, there are several variants of the cascading deficit theory—one is the magnocellular deficit theory of dyslexia, in which a low‐level impairment in the motion‐sensitive magnocellular pathway of the visual system is said to disrupt reading skill development (Stein, 2001, 2019; Stein & Walsh, 1997). Proponents of this theory have argued that sensitivity to transient sensory information may not be restricted to vision, but could also affect auditory processing (Stein & Talcott, 1999; Van Ingelghem et al., 2001; Witton et al., 1998). Hypothetically, auditory insensitivity to rapid cues could diminish an individual's ability to learn the sounds of their language, and hence develop PA.

Distinct from these sensory processing theories, proponents of the statistical‐learning hypothesis argue that a domain‐general deficit in sensory learning and perceptual decision‐making more broadly could explain why people with dyslexia perform poorly on myriad psychophysical tasks (Ahissar, 2007; Nicolson & Fawcett, 2018; Ziegler, 2008). It also purports to explain why some children struggle to learn the mapping between letters and sounds; if sensory information cannot be effectively used, then acquiring sensitivity to the regularities of language may be challenging. Despite interest in cognitive deficits at the level of abstracting sensory information, an exact mechanism is not agreed on; variants include an inability to appropriately adjust sensory decision‐making criteria (Lieder et al., 2019) and abnormal neural dynamics (Jaffe‐Dax et al., 2017; Krause, 2015; Perrachione et al., 2016). Despite differences in the details, what is consistent across these “non‐sensory” theories is that they posit that a more general deficit disrupts both (1) performance on experiments requiring a subjects to make a decision based on sensory information and (2) the development of reading skills.

Today, the literature remains inconclusive for several reasons. First, various cascading deficit models contradict one another as each posits distinct mechanisms for disrupting phonological processing. While a statistical learning model could potentially explain why so many struggling readers also perform poorly on visual psychophysics, it has not been established whether these two types of deficits co‐occur in the same individuals. The widespread use of group‐level statistics makes it challenging to interpret how many individuals show a given pattern of low‐level deficits, and the few studies focusing on individual patterns across a battery of diverse tasks do not encourage much hope for a uniform profile (Amitay et al., 2002; Ho et al., 2002; Menghini et al., 2010; Ramus et al., 2003; White et al., 2006).

Perhaps more importantly, it remains challenging to understand what relationship predictors from psychophysical tasks have with phonological predictors in determining reading ability—in other words, whether the influence of low‐level sensory processing mechanisms on reading skill is mediated by phonological processing. Talcott, Witton, et al. (2000) may have best addressed this by administering a battery of auditory, visual, and phonological tasks, concluding that a measure of visual motion processing explained some additional variance in reading skill beyond a measure of PA. A follow‐up study replicated the finding that visual and auditory psychophysics explained unique variance in both phonological and literacy skills but did not clarify the fit of a cascading model (Talcott et al., 2002). Several others have observed evidence that auditory and visual processing measures influence reading skill separate from the proposed phonological pathway (Snowling et al., 2019; Stein, 2001; White et al., 2006). Despite these findings, cascading deficit models remain at the forefront of the dyslexia debate, particularly for theories that hold a central role for sensory processing deficits (reviewed in Goswami, 2015).

There are several reasons why studies such as Talcott et al.’s are well‐cited, but not broadly adopted as conclusive evidence about sensory processing in dyslexia. In the last two decades, there has been growing focus on non‐sensory mechanisms that may affect how struggling readers perform on psychophysical tasks—a confound that many studies may not have sufficiently accounted for (Banai & Ahissar, 2004, 2006; Ramus & Ahissar, 2012). Furthermore, in the multifactorial literature, it is increasingly accepted that at least two dissociable aspects of phonological processing (PA and RAN) contribute to reading skill (Pennington et al., 2012; Wolf & Bowers, 1999, 2000). Previous work only explores the relationship of sensory measures to a single dimension of PA (Bosse et al., 2007; Talcott, Hansen, et al., 2000; Talcott et al., 2002; Zoubrinetzky et al., 2014). As evidence mounts that PA alone is unlikely to explain many (Snowling, 2008; Snowling & Melby‐Lervåg, 2016), or even most (Pennington et al., 2012) cases of dyslexia, it remains worth considering how individual differences in visual motion processing, or perceptual decision‐making more generally, will fit into changing conceptions of reading disability.

To separate the contributions of sensory encoding of visual motion from non‐sensory aspects of the decision‐making process, we revisit a widely used measure of visual motion sensitivity (random dot motion discrimination) with a mathematical modeling approach. The drift diffusion model (DDM) estimates the generating function that corresponds to an individual's pattern of responses and reaction times on a task (Ratcliff & McKoon, 2008), and has been used to understand how cognitive mechanisms associated with aging (Ratcliff, Thapar, et al., 2004), Attention Deficit Hyperactivity Disorder (ADHD) (Huang‐Pollock et al., 2017), and development (Ratcliff et al., 2012) manifest in psychophysical task performance. The model has been extensively used to describe decision‐making on the motion discrimination task (Gold & Shadlen, 2007; Palmer et al., 2005; Shadlen et al., 2013), and validated by electrophysiological work in non‐human primates (Shadlen & Newsome, 2001). As such, the DDM provides a rigorous way to explore the intersection of sensory integration and decision‐making in relation to reading skill. To date, this model has only been used to study reading disability in two studies of lexical decision‐making (Ratcliff, McKoon, et al., 2004; Zeguers et al., 2011).

Here we present two studies testing core‐deficit and multifactorial models of dyslexia; in Study 1, we asked how well measures of phonological processing (PA and RAN) can account for diagnoses of reading disability. For maximal statistical power, and to ensure consistency in diagnostic criteria, we utilize a large public dataset of hundreds of school‐aged children who have been undergone a standardized assessment by a panel of clinicians. In a core‐deficit model of dyslexia with a central phonological component, we would hypothesize that most children would be well‐classified according to standard phonological processing measures. Alternatively, the extent to which dyslexia occurs in children with high scores on phonological measures indicates that additional, unmeasured factors are important for understanding those children's reading difficulties.

In Study 2, we explore relationships between measures of phonological processing (PA and RAN), visual motion processing and perceptual decision‐making, estimated with the DDM (*N* = 106 school‐age children tested in our lab). With this dataset, we first investigate patterns of correlations between visual processing, cognitive and reading measures, and then test the hypothesis that a multifactorial model, in which both phonological and visual processing factors contribute independently to reading skill, outperforms any of the core‐deficit models.

## 2 METHODS: STUDY 1

### 2.1 Participants

The Healthy Brain Network dataset is provided to the public by the Child Mind Institute. At the time of writing, the released dataset included 1814 subjects. From this dataset, we identified 124 school‐aged individuals (ages 5–17) in the urban New York City region who had been diagnosed with “Specific Learning Disorder with Impairment in Reading” by a panel of clinicians affiliated with the Child Mind Institute and also had standardized scores on the Comprehensive Test of Phonological Processing (CTOPP‐2; Mitchell, 2001) available. The diagnoses were made according to the 5th edition of the Diagnostic and Statistical Manual for Mental Disorders (though specific criteria are not provided; American Psychiatric Association, 2013).

We also identified 119 individuals who were similarly assessed and given no diagnosis of any kind. Due to the large number of participants available, we were able to create nonverbal IQ matched control groups on the basis of the Wechsler Intelligence Scale for Children's Matrix Reasoning scaled score (Dyslexia: *n* = 110; Control: *n* = 105). These groups did not significantly differ in terms of nonverbal IQ (*t*(208.85) = −1.0668, *p = *0.287) or age (*t*(212.65 = 1.041, *p = *0.299). The Healthy Brain Network dataset can be accessed here: http://fcon_1000.projects.nitrc.org/indi/cmi_healthy_brain_network/index.html

### 2.2 Measures

We analyzed two standardized measures administered to all children in the Control and Dyslexia groups: the CTOPP‐2’s Elision subtest and the RAN Composite score. These age‐normed measures give an estimate of PA and RAN respectively.

### 2.3 Analysis

To assess the separability of the Control and Dyslexia groups on the basis of two measures of phonological processing, we used quadratic discriminant analysis (QDA). Using the QDA function from the MASS library for R, we fit a model with group as the dependent variable and PA and RAN as independent variables. To avoid overfitting, we report results from leave‐one‐out cross validation.

## 3 RESULTS: STUDY 1

### 3.1 Predicting dyslexia from phonological measures

**FIGURE 1:**
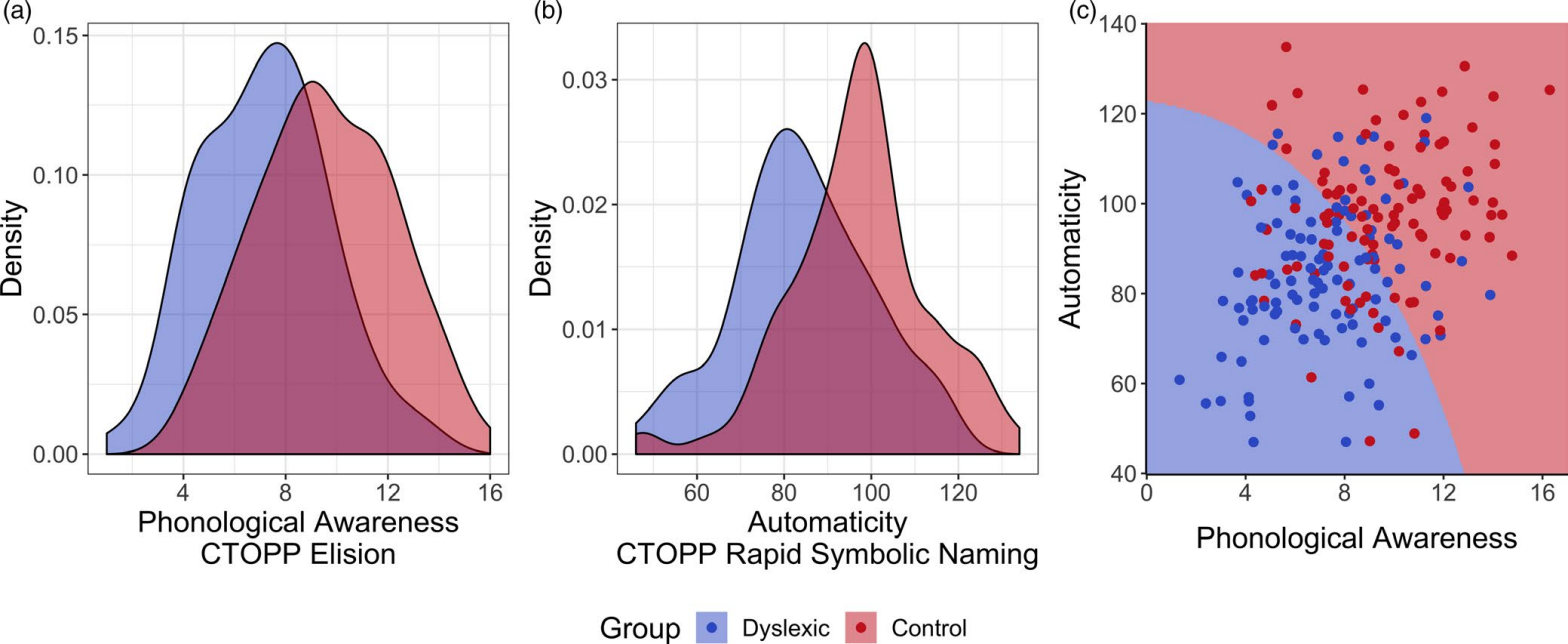
(a,b) Density plots for phonological awareness (Comprehensive Test of Phonological Processing [CTOPP] Elision) and rapid automatized naming (CTOPP Rapid Symbolic Naming Composite) in the Healthy Brain Network dataset in two subsets. The Dyslexia group (blue) consists of 110 school‐aged children diagnosed with dyslexia by a panel of clinicians. The red density plot represents an age‐ and nonverbal IQ‐matched control group of 105 children identified as having no psychiatric or neurological diagnoses by the same panel. (c) The decision boundary of a quadratic discriminant analysis trained on the entire dataset is shown. Dots represent observations from the dataset with slight jitter added for visibility of overlapping points

We first assessed the phonological core deficit model by quantifying the extent to which deficits in PA, RAN, or both differentiate individuals with dyslexia from control subjects with typical reading skills (Figure 1). A classifier trained with leave‐one‐out cross validation on both features could correctly classify 67.4% (±6.3%; 95% confidence interval) of individuals with a specificity of 68.2% and a sensitivity of 66.7%. To be certain that this does not reflect the limitations of a specific classifier model (QDA), we also assessed a support vector machine model and found no improvement in classification accuracy.

The classifier results are undoubtedly in alignment with the extensive literature on phonological processing; PA and RAN are both meaningful predictors of reading skill. As is clear in Figure 1a,b, there are pronounced group‐level differences on both measures; dyslexic and control groups differ by nearly a standard deviation on both PA and RAN measures. A dis‐attenuated estimate of Cohen's *d*, accounting for the published test–retest reliability of each measure, was 1.00 for PA (unadjusted *d* = 0.93) and 0.87 for RAN (unadjusted *d* = 0.81). Yet, these two measures alone fail to account for many cases of dyslexia—in the Healthy Brain Network (HBN) sample, 33 out of 110 cases (Figure 1c). For either measure, there would be approximately 50% overlap between Control and Dyslexic groups even when accounting for measurement reliability, and many individuals with apparently typical reading abilities would be erroneously predicted to be dyslexic based on their PA and RAN scores alone (low specificity).

In the original formulation of the phonological core deficit model (e.g., Stanovich, 1988), PA is purported to be a more powerful predictor of reading disability in early childhood, so it may be unsurprising that the model's accuracy is not higher in a sample containing teenagers. We therefore repeated the analysis on two subsets of the sample: 62 children between ages 5 and 8 (*n* = 29 with a Dyslexia diagnosis), and 153 children aged 8–17 (*n* = 81 with a Dyslexia diagnosis). The classifier trained on the younger cohort obtained an accuracy of 69.4% (±11.7%) while the classifier trained on the older cohort reached 66.7% (±7.5%). We ran a second analysis treating age as a continuous predictor and we used logistic regression on our entire sample to model dyslexia diagnosis (present or absent) with main effects of age, PA, and the interaction of the two. The interaction term was not significant (*β* = −0.007, SE = 0.0243, *p* = 0.767), indicating that the predictive value of PA and RAN was roughly consistent across the sampled age range.

Finally, we tested a direct measure of pseudoword reading skill provided in the HBN dataset (the age‐normed Weschler Individual Achievement Test Pseudoword subtest) as the dependent variable in a linear model. The interaction of age and PA was again not significant (*β* = −0.0322, SE = 0.116, *p* = 0.782). Similarly, the interaction of age and RAN was not significant (*β* = −0.0357, SE = 0.019, *p* = 0.070). As such, our finding that standard phonological measures are modest, yet imperfect, predictors of dyslexia in the HBN dataset is unlikely to be an artifact of the age range of the sample.

## 4 STUDY 2

Having demonstrated that phonological predictors alone are insufficient to accurately distinguish many cases of dyslexia from typical reading (Study 1, HBN data), we next consider the contribution of visual motion processing to reading abilities. Do visual motion processing difficulties typically coincide with phonological impairments, as would be expected in a cascading model of reading disability? Or are they a separable contributor to reading outcomes which explain cases of dyslexia that were not captured by the phonological core deficit model? Here we present the results of the motion discrimination experiment (conducted in the lab) in 106 school‐aged children, including 42 individuals who meet our criteria for dyslexia.

### 4.1 Methods

#### 4.1.1 Participants

A total of 119 native English‐speaking school‐aged children aged 8–12 were recruited. Children without histories of neurological or sensory disorders were recruited from a database of volunteers in the Seattle area (University of Washington Reading & Dyslexia Research Database; http://ReadingAndDyslexia.com). Parents and/or legal guardians of all participants provided written informed consent under a protocol that was approved by the University of Washington Institutional Review Board. All subjects demonstrated normal or corrected‐to‐normal vision.

Five subjects did not complete the psychophysics. An additional two subjects did not show evidence of performing above chance (>60.5% accuracy at any of the four stimulus coherence levels) and were excluded from analysis. A further six subjects did not produce enough usable data to fit the DDM (no more than 15% responses outside of the acceptable response time window from 200 ms to 10 s). This left 106 subjects with usable data. The average age of these participants was 9.9 years (SD = 1.3).

#### 4.1.2 Measures of literacy and cognitive skills

Several standardized measures were used to assess foundational literary and cognitive skills in our participants. All participants completed the subtests of the Woodcock–Johnson IV (Schrank et al., 2014) required to estimate the Basic Reading Score (WJ‐BRS), Letter Word Identification, and Word Attack. To obtain the test of word reading efficiency (TOWRE) Index, participants completed the Sight Word Efficiency and Phonemic Decoding Efficiency subtests of the TOWRE‐2 (Torgesen et al., 2011). Phonological processing was assessed with the CTOPP‐2 (Mitchell, 2001). A PA score was obtained as a composite of the Elision, Blending Words, and Sound Matching subtests. A RAN score was obtained as a composite of the Rapid Digit Naming and Rapid Letter Naming subtests. Additionally, a Phonological Memory composite score was obtained as a composite of the Memory for Digits and Nonword Repetition tasks. Lastly, all participants completed the Weschler Abbreviated Scale of Intelligence‐II (Wechsler, 2011) Vocabulary and Matrix Reasoning subtests. A composite of these two scores yielded the Full Scale‐2 composite. The Matrix Reasoning score was used as a measure of nonverbal IQ in the following analyses.

#### 4.1.3 Definition of dyslexia and control groups

We recruited participants whose reading abilities ranged from profoundly impaired to highly proficient. Since reading abilities fall on a continuum (Shaywitz et al., 1992), and because we could not ensure that children in our area with parental reports of a diagnosis were diagnosed in a standardized way, we treat reading ability as a continuous measure in our main statistical analyses. For the purpose of comparison with other studies, we also include group‐level analyses (Dyslexic vs. Control). For sake of brevity, the complete group analysis is described in Supplementary Materials and we refer to key findings from this complimentary analysis in the main text where appropriate. Group labels were assigned on the basis of the composite WJ‐BRS and TOWRE Index. As both the WJ‐BRS and TOWRE Index are scored on the same standardized scale, a composite reading skill measure was created by averaging the two scores for each participant. The average reading score overall was 92.0 (SD = 19); note that this was significantly lower than the expected population mean of 100 (*t*(105) = −4.31, *p* < 0.001), indicating that poor readers were oversampled in our recruitment. The “Dyslexic” group comprised participants whose reading score fell 1 SD or more below the population mean (reading score <85); the “Control” group had reading scores above this cutoff and had never been diagnosed with a reading disability. There were 43 subjects in the Dyslexic group and 48 in the Control group. A remaining 15 subjects were not well‐described by either label (e.g., reading score >85 but an indication of a dyslexia diagnosis) so were not included in the group comparisons. As in several other studies (O’Brien et al., 2018; Pennington et al., 2012), we did not IQ‐match these groups, but rather controlled for nonverbal IQ explicitly in our statistical analyses. Additionally, ADHD diagnosis was not grounds for study exclusion because of the high comorbidity between ADHD and dyslexia. The presence of ADHD was entered into our linear modeling analyses as a covariate. Relationships between demographic characteristics, phonological, IQ measures, and reading skill are presented in Tables S1 and S2.

#### 4.1.4 Psychophysics stimuli and apparatus

Participants were tested with a motion discrimination paradigm, a single‐interval task in which participants are asked to label the overall direction of motion (left or right) for a patch of random‐dot motion stimuli generated with varying coherence levels. When coherence is at 0%, participants perform at chance levels, as there is effectively no signal. Also, as the coherence is increased, so too increases the salience of perceived motion to the left or right. For further details of the stimuli and testing apparatus, please see Supplementary Methods.

#### 4.1.5 Psychophysics procedure

Each session comprised six experimental blocks. For each subject, three blocks of 50 stimuli were tested with a brief break in between. This was followed by a longer break to collect reading, phonological and IQ measures, and followed by the final set of three blocks. At the beginning of the session, subjects completed 10 practice trials comprising high coherence motion (60%–100%). Subjects were allowed to repeat the practice up to three times, until they got at least 70% correct. All participants were able to do this.

Stimuli were presented at five coherence levels: 6%, 12%, 24%, 48%, and 100%. However, early in the study we realized that many subjects (unrelated to reading ability) found 100% coherence difficult and reported varying visual percepts. Performance typically declined for 100% coherence stimuli compared to 48% coherence. Therefore, we analyzed only the range of stimulus coherence levels where performance was generally monotonic, from 6% to 48%. Each stimulus coherence level was presented 60 times for a total of 300 presentations, 240 of which were included in the analysis.

Each trial started with a fixation mark at the center of the display. After 500 ms, random‐dot motion stimuli were displayed until the subject made a keypress (or until 10 s had elapsed). Subjects pressed right or left arrow keys on a standard keyboard to report motion direction. The fixation mark was turned off when the response was made, and visual and auditory feedback was given to indicate correct and incorrect responses. The experiment did not proceed until subjects reported the motion direction. The inter‐trial interval was 1 s, and after this interval the fixation mark re‐appeared at the center of the display to indicate the beginning of the next trial.

#### 4.1.6 Drift diffusion model

To decouple sensory encoding of visual motion from the process of forming and executing a binary decision, we fit the DDM to each subject's distribution of behavioral responses and reaction times. In the DDM for a two‐alternative forced‐choice judgment, it is assumed that an observer samples sensory input at discrete moments in time, and that these samples are accumulated in a noisy decision variable that represents the integrated evidence over the course of the trial (plus internal noise). When this decision variable reaches a threshold, the observer initiates a decision. The DDM therefore separates the encoding and evaluation of sensory information (which drives changes in the decision variable) from non‐sensory processes, such as the magnitude of the threshold for triggering a decision and the trial‐to‐trial variability in the decision process (for a detailed review of the DDM, see Ratcliff & McKoon, 2008; Wiecki et al., 2013).

For further details of the DDM implementation, outlier detection, and modeling procedure for testing hypotheses around DDM parameters, see Supplementary Methods.

### 4.2 Results: Study 2

#### 4.2.1 Visual motion processing and reading abilities

Before we model the respective contributions of sensory and decision processes to task performance, it is important to establish that task performance is related to reading skill. We confirmed that reading skill was related to reaction time; using model selection, we identified that the most parsimonious model of median reaction time included main effects of stimulus coherence (*β* = −0.173, SE = 0.00898, *p* < 1 × 10^−15^), age (*β* = −0.059, SE = 0.0214, *p* < 1 × 10^−15^), and reading skill (*β* = −0.006, SE = 0.00149, *p = *1.15 × 10^−4^) with a random effect of subject (Table S4; Figure S1). Accuracy was not significantly related to reading skill (Table S5), likely reflecting the fact that the motion stimuli remained on the screen until the subject provided a response. Notably, we also observed that the ratio of correct to error median response times within each subject was significantly associated with reading skill (*β* = −0.00444, SE = 0.00224, *p = *0.0497), with poor readers showing an increased tendency to make “fast errors” relative to correct response times (Table S6; Figure S2). The presence of fast errors is notable because this phenomenon is typically associated with non‐sensory mechanisms, including a tendency to initiate guesses before an optimal amount of evidence is considered (Smith & Ratcliff, 2004). Thus, raw reaction time data indicated that children with low reading scores were not only less efficient than control subjects in processing visual motion, but also showed a qualitatively different pattern of responses.

#### 4.2.2 Less efficient visual motion processing in children with low reading scores

After fitting the DDM to each subject's behavioral responses, we investigated whether there was a relationship between the drift rate parameter, *v*, and reading skill. Drift rate models the efficiency with which information is extracted and integrated from incoming sensory signals. For example, drift rate monotonically increases with stimulus coherence level (*β* = 0.719, SE = 0.0249, *p* < 1 × 10^−15^) indicating the visual system can more efficiently extract motion information when stimulus noise is low. If individuals with dyslexia do not have any difficulties with encoding visual information, as predicted by the statistical learning hypothesis, we would expect drift rate to be uncorrelated with reading skill once covariates like IQ, age, and ADHD diagnosis are controlled for. Note that in our analyses, we treat reading as a continuous measure, but we also provide analyses where reading disability is treated as a categorical variable in Supplementary Analysis 1 (Tables S7–S9). Details of the model selection procedure are provided in the Methods.

**FIGURE 2:**
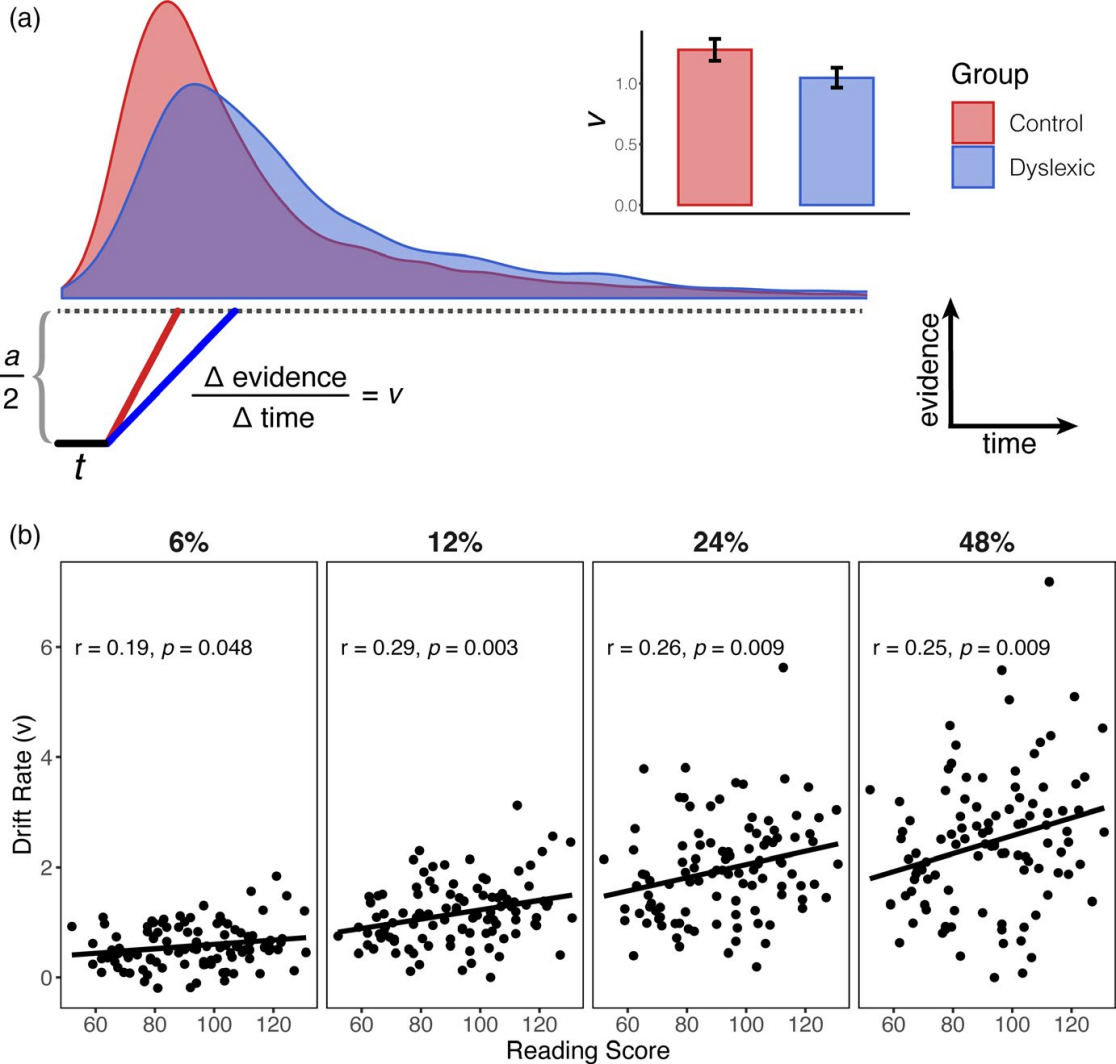
(a) A schematic of the drift diffusion model (DDM) with reaction time distributions (at 12% coherence) from the control and dyslexic groups imposed above. The red and blue lines in the schematic show how differences in drift rater predict differences in the reaction time distributions. The DDM model was fit separately to each individual's data and the average drift rate parameter for the dyslexic and control groups is shown in the bar plot in panel a (±1 SE). (b) The relationship between estimated drift rate and reading skill at four different stimulus coherence levels. Lines are best fit regression lines and shaded regions are confidence intervals

Individual estimates of drift rate are shown in Figure 2. Drift rate was best modeled by a main effect of reading skill, a main effect of stimulus coherence, a main effect of age, and the interaction of reading skill and stimulus coherence (Table 1). Our results therefore indicate that drift rate increases with stimulus coherence, as expected, as well as age and reading skill. Furthermore, there is a stronger relationship between reading skill and drift rate at high stimulus coherence levels, which is likely a consequence of the fact that estimates of drift rate are more reliable at higher coherence levels (see Methods).

**TABLE 1:** Selected model of drift rate

	*β*	SE	*p*
Intercept	1.534	0.0620	<1 × 10^−15^
Stimulus coherence	0.719	0.0249	<1 × 10^−15^
Age	0.268	0.0623	3.878 × 10^−5^
Reading skill	0.173	0.0623	6.605 × 10^−3^
Stimulus coherence: Reading skill	0.0869	0.0249	7.04 × 10^−4^

To estimate the effect size of the relationship between reading skill and drift rate, we considered a linear model of reading skill as a function of participant's average drift rate; the selected model of reading skill contained main effects of mean drift rate (*β* = −0.262, SE = 0.009, *p* = 0.006) and nonverbal IQ (*β* = −0.488, SE = 0.008, *p* = 5.20 × 10^−8^). In this model, the partial *r* ^2^ associated with mean drift rate was 0.074; thus the unique contribution of this index of visual motion processing to explaining variance in reading skill is likely modest. Similarly, with reading skill treated as a group‐level variable, Cohen's *d* was 0.42. As such, our data do not provide evidence that deficits in motion processing occur in most struggling readers, though there is a significant relationship between motion processing and reading ability.

**FIGURE 3:**
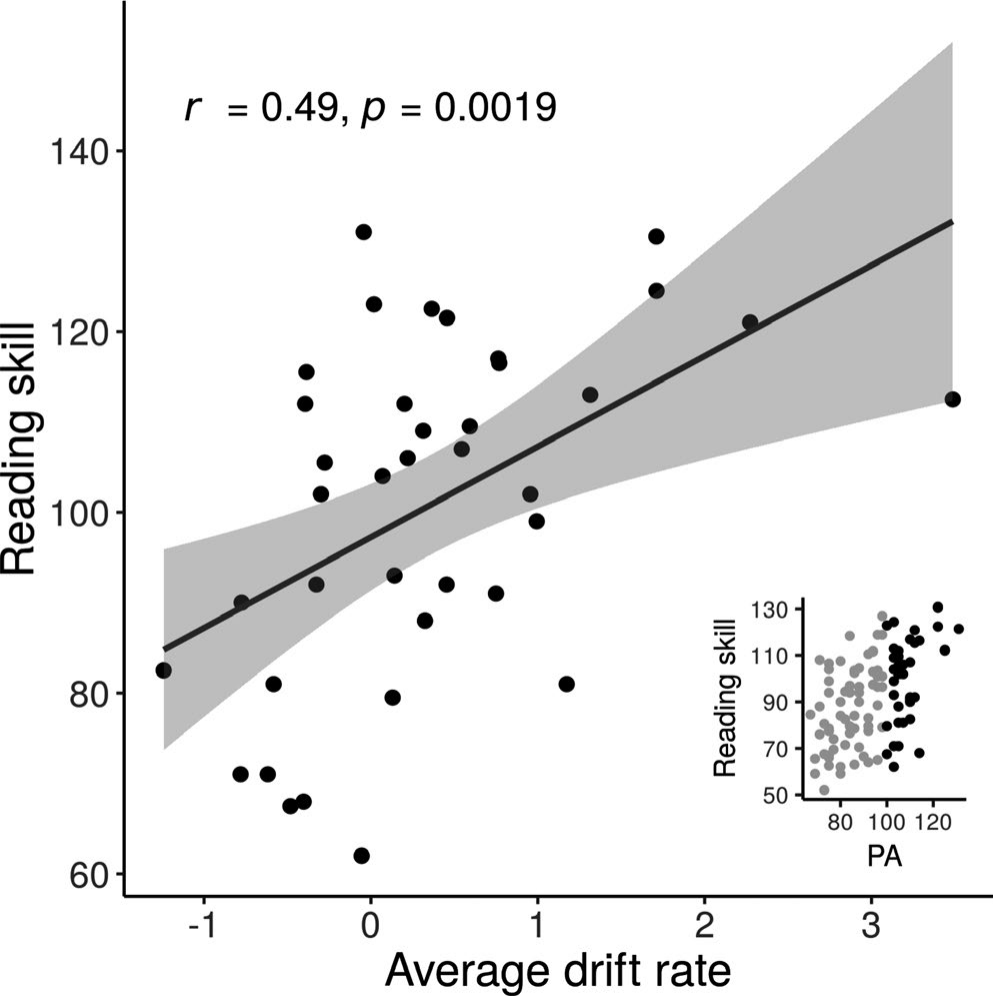
The relationship between drift rate and reading skill in a subset of individuals with good phonological awareness. Average drift rate is calculated by averaging each individual's *z*‐scored drift rate estimates at each stimulus coherence level. Inset: a scatter plot indicating in black which subset of the study sample is included in the “good phonological awareness” group

As to the question of whether drift rate explains additional variance in reading skill beyond phonological processing, consider the subset of readers in our sample with above average PA (PA scores ≥100). Within this subgroup of 38 participants, 9 children (23.7%) met our criteria for dyslexia despite having high PA, and reading skill was significantly correlated with mean drift rate (*r* = 0.49, *p = *0.0019; see Figure 3). The relationship between reading skill and mean drift rate in the high PA subgroup remained significant when controlling for RAN and nonverbal IQ (*β* = −0.164, SE = 0.072, *p* = 0.0279). For these individuals, knowing drift rate explains 24% of variance in reading skill. In readers with average‐or‐better PA, it appears that individual differences in visual motion processing distinguish between struggling and expert readers. Interestingly, a different pattern emerged in a subgroup of 28 subjects with RAN scores ≥100: for these participants, the correlation between reading skill and drift rate was not significant (*r* = 0.03, *p* = 0.320).

#### 4.2.3 Decision‐making parameters are related to reading skill and independent of visual processing

**FIGURE 4:**
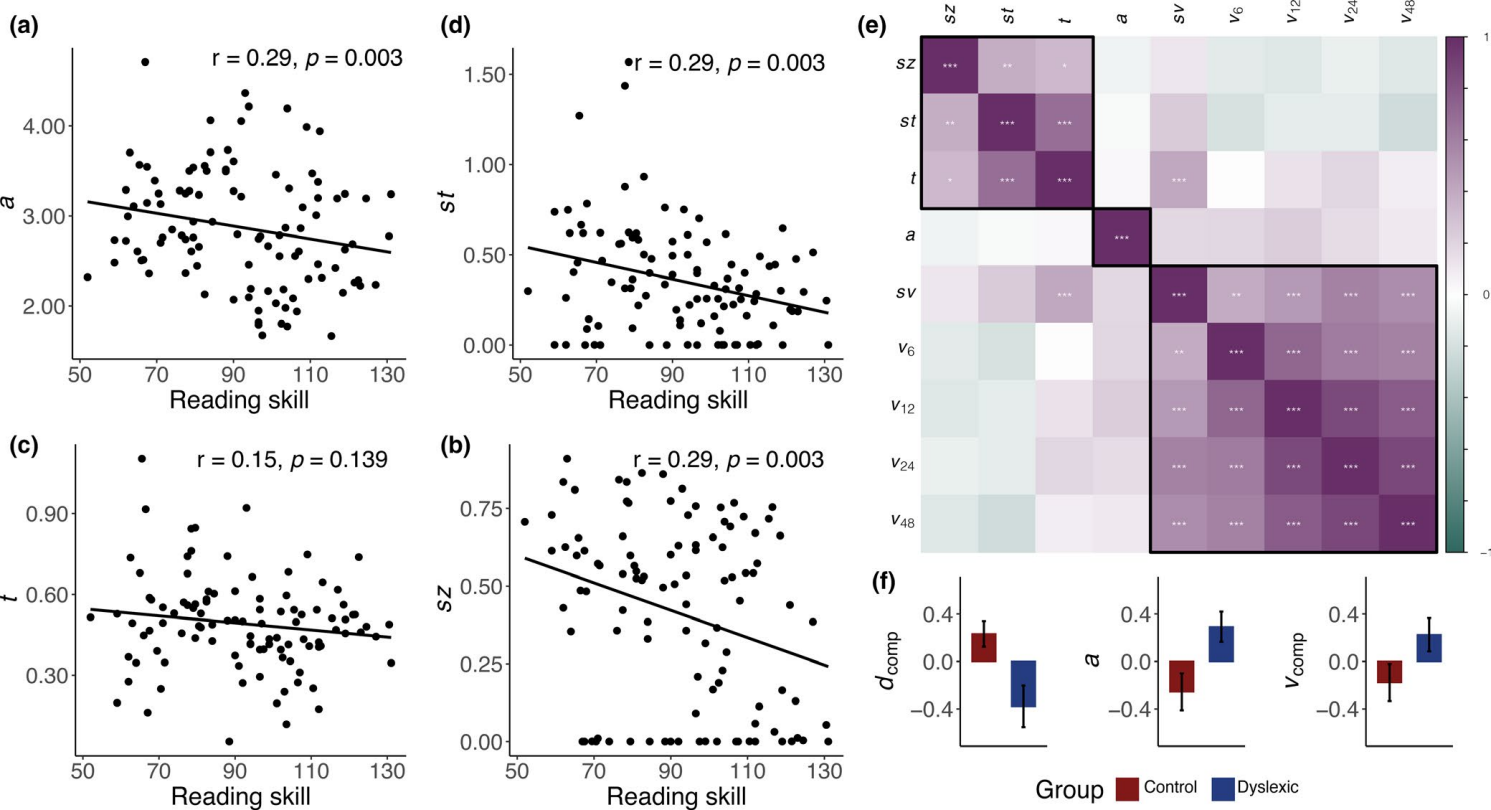
(a–d) The relationship between reading score and four non‐sensory parameters of the drift diffusion model (DDM). (a) decision threshold *a*, (b) variability in drift process starting point *s_z_*, (c) non‐decision time *t*, and (d) variability in non‐decision time *s_t_*. (e) Correlations between parameters of the DDM. Boxes indicate hierarchical clustering results (Ward's method) and stars indicate significant correlations after Holmes–Sidak correction for multiple comparisons: *p < 0.05, **p < 0.01, and ***p < 0.001. (f) Group comparisons for the three composite measures based on hierarchical clustering of the DDM parameters: *d*_comp_: composite of *s_z_*, *s_t_*, and *t*, the *a* parameter, and *v*_comp_: composite of the four drift rate parameters and *s_v_*. Note that all three composite parameters are *z*‐scored. Error bars represent 1 SEM

We next consider the predictions of non‐sensory hypotheses by analyzing the relationship between parameters of the DDM that index non‐sensory components of the decision‐making process and reading skill (Figure 4a–d). For each parameter of interest, we modeled reading skill as a function of the parameter plus the covariates and report the results of model selection. If poor readers struggled with the task only because of differences in visual processing, we would expect no parameters besides drift rate (and *s_v_*) to be useful predictors of reading skill.

To the contrary, the parameter *s_z_* was significantly correlated with reading skill and, after model selection, was retained as a predictor (Figure 4a). The selected model contained main effects of *s_z_* (*β* = −0.798, SE = 0.281, *p = *0.005) and nonverbal IQ (*β* = 0.483, SE = 0.083, *p = *7.05 × 10^−8^). The parameter *s_z_* represents the trial‐to‐trial variability in the relative amount of evidence required to initiate a judgment; individuals with high *s_z_* values are prone to making fast errors. Indeed, we confirmed that the ratio of median correct response times to error response times within a subject was correlated with the DDM estimation of *s_z_* (*r* = 0.452, *p = *1.44 × 10^−6^).

Similarly, we observed that the parameter representing the threshold of evidence required to initiate a decision, *a*, had a modest but significant correlation with reading skill (*β* = −0.136, SE = 0.0632, *p = *0.0329), indicating that worse reading skill is associated with employing a more conservative criterion for initiating a perceptual decision (Figure 4b). After model selection, *a* and nonverbal IQ were retained as selectors of reading skill (*β* = −0.237, SE = 0.130, *p = *0.072; *β* = 0.490, SE = 0.085, *p = *8.79 × 10^−8^).

Lastly, we examined parameters that represent the lumped contributions of all non‐decision processes to reaction time, including the time necessary to encode a visual stimulus and execute a motor response. Because some individuals with dyslexia are known to have slower processing speed (Pennington et al., 2012; Peterson & Pennington, 2015), we might expect this time to be longer in children with worse reading skills. Indeed, the parameter *t* representing an individual's average non‐decision time showed an overall negative relationship with reading skill; model selection retained both *t* (*β* = −1.031, SE = 0.489, *p = *0.0375) and nonverbal IQ (*β* = 0.518, SE = 0.841, *p = *1.50 × 10^−8^; Figure 4c). We also tested a model of reading skill as a function of a parameter modeling trial‐to‐trial variability in non‐decision time, *s_t_* (Figure 4d). Model selection retained both *s_t_* (*β* = −0.960, SE = 0.266, *p = *0.0004) and nonverbal IQ (*β* = 0.508, SE = 0.0807, *p = *7.89 × 10^−9^).

We have so far identified several parameters of the DDM indexing both visual and non‐sensory processes that show univariate associations with reading skill (even after covariates for age, nonverbal IQ, and ADHD diagnosis are considered). We next considered the extent to which these parameters were correlated with one another, potentially indicating clusters of parameters that index a common underlying mechanism (Figure 4e). As expected, we noted strong correlations between the four drift rate parameters. None of the drift rate parameters were significantly correlated with any non‐sensory parameters after correction for multiple comparisons. There were moderate correlations between three non‐sensory parameters, *s_t_*, *t*, and *s_z_* (*s_t_* and *t*: *r* = 0.685, *p = *9.75 × 10^−16^; *t* and *s_z_*: *r* = 0.335, *p = *0.0005; *s_z_* and *s_t_*: *r* = 0.386, *p = *5.03 × 10^−5^). These three parameters largely contribute to modeling the leading edge of the reaction time distribution—*s_z_* allows for the presence of relatively fast errors, *t* shifts the response time distribution along the time axis, and *s_t_* allows for responses before an individual's average response time. Finally, we noted that the parameter *a* was uncorrelated with any of the other parameters. Hierarchical clustering (Ward's method, Ward, 1963) indicated that the correlation matrix was consistent with three clusters of parameters: a cluster consisting only of *a*, another consisting of the *s_t_*, *t*, and *s_z_*, and a final cluster including all four drift rates and *s_v_*. This suggests that the DDM captures several distinct mechanisms underlying visual encoding and perceptual decision‐making. The correlation matrix of all DDM parameters and three hierarchical clusters are diagrammed in Figure 4e.

#### 4.2.4 Visual and non‐sensory predictors both explain reading outcomes

So far in our analysis, there seem to be several separate profiles of performance on the motion discrimination task that are associated with low reading skill: (1) reduced ability to encode and integrate visual information, (2) setting a more conservative decision criterion, and (3) generally more variability in terms of the time taken to gather evidence and/or execute a decision. The lack of correlations between many of the DDM parameter estimates indicates that individuals who display a deficit in terms of one process (e.g., visual encoding), are not necessarily the same individuals who perform abnormally in terms of another process (e.g., decision‐making), and that profiles of performance are variable across subjects. To test whether each dimension of task performance is indeed a unique contributor to a model of reading skill, we employed a linear modeling approach (with reading skill as the dependent variable). To simplify the number of parameters, we introduce several composite measures based on our clustering analysis (Figure 4e). Drift rate is summarized as a composite measure, *v*_comp_, by taking the first principal component of the four drift rates and *s_v_*. A second composite measure *d*_comp_ was derived from the first principal component *s_t_*, *t*, and *s_z_*, which we expect represents aspects of variability in the decision‐making process.

We performed model selection, starting with the full model with reading score as the dependent measure and all hypothesized DDM parameters and the three covariates (*v*_comp_, *d*_comp_, *a*, nonverbal IQ, ADHD diagnosis, and age) as predictors. The selected model retained all three predictors from the DDM and nonverbal IQ (Table 2). This result confirms that non‐sensory mechanisms explain additional variance in reading skill once the quality of visual encoding is accounted for. As such, even within this single psychophysical task, there are multiple non‐correlated dimensions of variance contributing to the pattern of responses observed in individuals with dyslexia—the ability to extract evidence from visual information, choice of decision threshold, and trial‐to‐trial variability in behavior.

**TABLE 2:** Selected model of reading skill from drift diffusion model parameters

	*β*	SE	*p*
Intercept	0.972	0.351	0.00663
*v*_comp_	−0.274	0.0778	6.56 × 10^−4^
*a*	−0.339	0.119	0.00548
*d*_comp_	0.291	0.0755	2.10 × 10^−4^
Nonverbal IQ	0.0453	0.0766	4.68 × 10^−8^

#### 4.2.5 Do sensory deficits have cascading effects?

To address the question of whether performance on the motion discrimination task is related to reading skill by way of phonological processing, or in addition to it, we explore a series of linear models. We first test the hypothesis that predictors from the psychophysical task do not explain additional variance in reading skill once phonological processing is accounted for. We again modeled reading skill as a function of composite measures from the DDM—*v*_comp_, *d*_comp_, and *a*—as well as two phonological processing measures, PA and RAN, and the three covariates. Model selection retained all predictors except ADHD diagnosis and age (Table 3). Correspondingly, an ANOVA *F*‐test comparing the selected model to a reduced model with only PA, RAN, and nonverbal IQ confirmed that adding predictors from the DDM explained variance in reading skill above and beyond the reduced model (*F*(100, 97) = 4.0438, *p = *0.00936). The reduced model also had a higher Akaike Information Criterion (AIC) and Bayesian Information Criterion (BIC) (selected model AIC = 794.4 and BIC = 813.9; reduced model AIC = 800.7 and BIC = 815.6). Because ordinary least squares models may be poorly affected by multicollinearity, we also applied lasso regression with 10‐fold cross validation (Friedman et al., 2010), and confirmed the same finding (modeling approach is provided in Figures S3 and S4; Table S10). Lastly, mediation analyses revealed, at most, a partial mediation effect for PA on the relationship between DDM parameters and reading skill (see Supplementary Analysis 2). From these analyses, we can confirm that all three predictors from the DDM are useful for explaining differences in reading skill *in addition* to traditional measures of phonological processing.

**TABLE 3:** Selected model of reading skill

	*β*	SE	*p*
Intercept	−0.554	0.256	0.0331
*v*_comp_	−0.120	0.0601	0.0491
*a*	−0.193	0.0871	0.0293
*d*_comp_	0.142	0.0570	0.0140
Nonverbal IQ	0.335	0.0602	2.26 × 10^−7^
CTOPP PA	0.172	0.0653	0.0097
CTOPP RAN	0.521	0.0582	2.49 × 10^−14^

Abbreviations: CTOPP, Comprehensive Test of Phonological Processing; PA, phonological awareness; RAN, rapid automatized naming.

#### 4.2.6 Multiple dimensions of skilled and disabled reading

**FIGURE 5:**
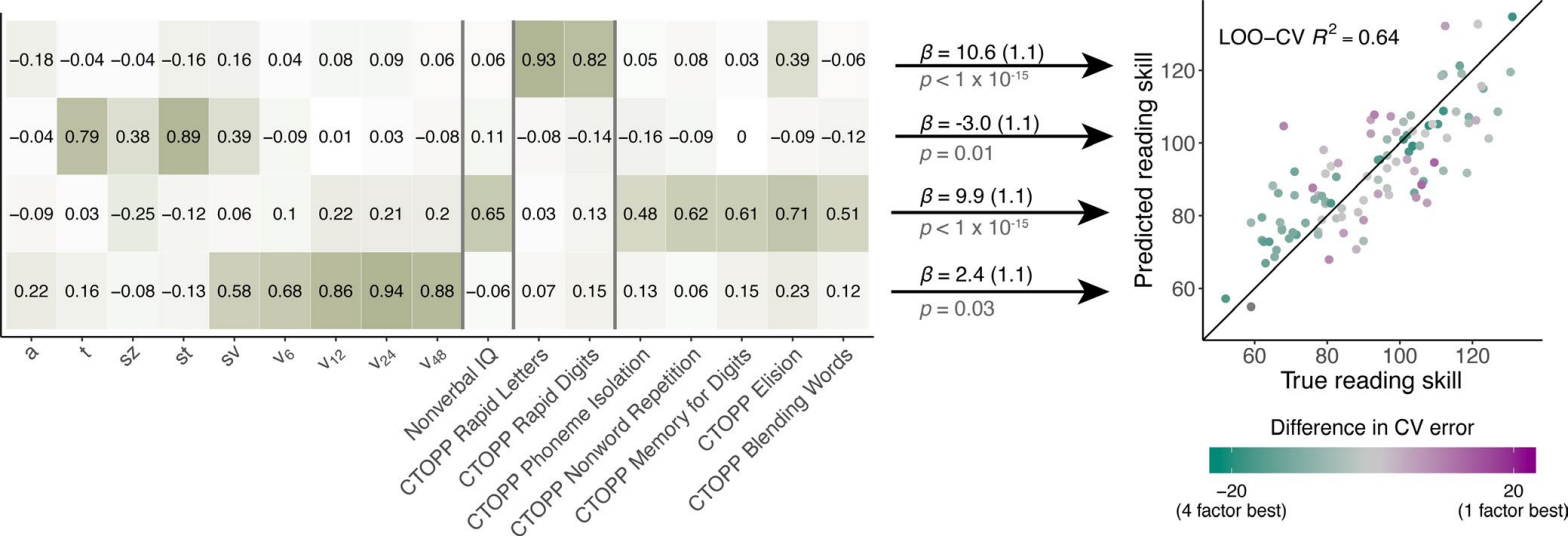
Factor loadings for the orthogonal four‐factor model are shown in the table; shading corresponds to absolute value of the loading. The scatterplot shows the correspondence between true (measured) and predicted reading skill using a linear model with all four factors as predictors. Each point was predicted using leave‐one‐out cross‐validation (LOO‐CV). Color indicates whether that point was more accurately predicted by the single‐factor model or the full model with all four factors. Green points had a lower squared error when predicted by the four‐factor model, and purple points had a lower squared error when predicted by the single‐factor. Gray points had similar prediction accuracy for both models. CTOPP, Comprehensive Test of Phonological Processing

Contrary to theories that seek to discover a unified deficit that characterizes reading difficulties, we have established that visual motion processing is separable from non‐sensory aspects of perceptual decision‐making, and both factors account for independent variance in reading skill. To speak to the question of how many separable underlying factors predict reading skill, we next apply exploratory factor analysis (EFA), an unsupervised learning approach for identifying the number, and characteristics, of *latent factors* that explain the correlation structure of a dataset (Costello & Osborne, 2005; Ferguson & Cox, 1993; Kline, 2013). We applied EFA to characterize the space of the DDM parameters, nonverbal IQ, and the six subtests of the CTOPP. An analysis of the eigenvalues of the correlation matrix indicated that four latent factors were warranted (i.e., the first four eigenvalues >1, see scree plot in Figure S5), and this was confirmed by parallel analysis (Hayton et al., 2004). The four factors are shown in Figure 5 with orthogonal varimax rotation. The total proportion of explained common variance by the four‐factor model was 55.8% (Factor 1: 20.3%, Factor 2: 14.2%, Factor 3: 10.7%, Factor 4: 10.6%).

The loadings of the first factor are dominated by the four drift rate parameters, whereas the second factor is loaded most heavily by nonverbal IQ and four of the CTOPP subtests. The remaining two subtests, Rapid Digits and Rapid Letters, load onto their own factor (in line with the double‐deficit hypothesis, Wolf & Bowers, 1999). An additional factor appears to reflect non‐decision time and variability parameters of the DDM *s_t_*, *s_z_*, and *t*. Notably, the evidence threshold parameter, *a*, is not particularly associated with any factor; 87% of variance in *a* is unexplained by this model.

Factor analysis largely conforms to the intuitions we have built so far from linear models: drift rate, although correlated with phonological processing and perhaps partially mediated by it, is identified as a separate factor. Drift rate and the non‐sensory parameters of the DDM are modeled as observations from two distinct factors. It is likely that *a* is representative of an additional factor, consistent with its lack of correlations with any other parameter of the DDM (note that without multiple estimates of *a*, EFA cannot estimate measurement noise and consequently does not assign it to a new factor). Critically, each of these four factors was significantly related to reading skill consistent with the interpretation that, rather than representing a single underlying construct, there are multiple, independent cognitive and sensory dimensions characterizing individual differences in reading skill (Figure 5). A linear model of reading skill as a function of scores on the four factors indicated that all four effects were significant (see coefficients in Figure 5). Furthermore, the full model also had a lower AIC (full model AIC = 798.8, single factor model AIC = 869.9) and BIC (full model BIC = 814.6, single factor model BIC = 877.8).

In addition to standard model selection, we compared the accuracy of the four‐factor model on predicting held‐out observations to the accuracy of a single‐factor model. Using leave‐one‐out cross validation, the four‐factor model explained 63.9% of variance in reading skill for the held‐out points. The single factor model used only Factor 2, which is largely a composite of the CTOPP measures of PA, phonological memory, and nonverbal IQ. This model only explained 27.4% of variance in reading skill for held‐out observations (Figure S6), indicating the necessity of considering multiple (at least four) underlying dimensions in order to accurately predict individual differences in reading ability.

## 5 CONCLUSIONS

Our results demonstrate that (1) a core phonological deficit model is insufficient to account for many cases of developmental dyslexia, (2) abnormal performance on the motion discrimination experiment in children with dyslexia cannot be ascribed to a uniform profile of either visual processing or non‐sensory deficits, (3) both visual and non‐sensory mechanisms explain variance in reading skill above and beyond phonological processing, (4) the correlational structure of cognitive, linguistic, and visual measures explored here is consistent with, at minimum, four underlying factors, (5) each of these four factors accounts for unique variance in children's reading abilities. In sum, our results are not consistent with models of dyslexia that only consider phonological processing or models in which impairments in visual processing or decision‐making primarily affect reading development via a disruption of phonological processing. Instead, dyslexia should be conceptualized as a disorder that may arise from several distinct loci.

Our work is consistent with that of Pennington and colleagues, which has capitalized on large samples to demonstrate that individuals with dyslexia have a heterogeneous profile of cognitive and linguistic impairments (Pennington, 2006; Pennington et al., 2012; Peterson & Pennington, 2015). The present work extends this perspective to address the role of sensory processing and perceptual decision‐making deficits in dyslexia.

Several preceding studies have attempted to investigate multiple candidate mechanisms of dyslexia, including auditory, visual, and motor processes. Our work generally conforms to the finding of at least four such studies (Ho et al., 2002; Menghini et al., 2010; Ramus et al., 2003; White et al., 2006) that show a heterogenous pattern of deficits present in struggling readers. For example, Talcott *et al*. collected several psychophysical measures in 350 school‐aged children and, like us, found that each explained a small, but unique, percentage of variance in reading skill (Talcott, Witton, et al., 2000). Valdois and colleagues have argued that deficits in visual attention are independent of phonological processing deficits and represent a unique cause of dyslexia (Bosse et al., 2007; Lobier & Valdois, 2015; Lobier et al., 2012; Zoubrinetzky et al., 2014), but this point remains contentious for a variety of reasons (Saksida et al., 2016).

To our knowledge, the present work is the first use of the DDM to model motion discrimination in relation to reading skill. Our results from Study 2 serve as a partial validation of two seemingly contradictory theories: some poor readers show a pattern of performance consistent with reduced ability to extract information from incoming visual signals, while others are better described as having normal visual processing but altered decision‐making characteristics (including, as the propensity to make fast errors reveals, more trial‐to‐trial variability in the relative amount of evidence needed to initiate a decision). It is interesting to note that studies of lexical decision‐making have revealed similar differences in the decision‐making process (elevated evidence criteria) suggesting a potential link between performance on simple perceptual judgments (i.e., motion discrimination) and altered lexical access during reading (Zeguers et al., 2011). Neither the statistical learning hypothesis, which would argue that sensory deficits are not meaningful, nor the magnocellular deficit hypothesis, which would fail to predict the non‐sensory parameters of the DDM relate to reading skill, entirely match our results. Yet we see evidence for both visual‐ and non‐sensory differences in our sample. In line with these findings, we propose that each mechanism should be reconceptualized as a dimension of risk, as opposed to a single cause, of reading difficulties.

As a correlational study, our results cannot validate any particular causal mechanism. It is possible that each factor represents clusters of symptoms that indicate underlying abnormalities in a processing system, but are not a direct cause of reading difficulties themselves. For example, the fact that differences in visual motion processing predict unique variance in reading skill does not necessarily mean that, for those individuals, poor perception of visual motion is the cause of their reading difficulty. Instead, measurements of task performance may be a proxy for the fidelity with which the visual system constructs a sensory representation of a noisy stimulus (Sperling et al., 2005, 2006), or the efficiency of information transfer between different visual regions (Yeatman et al., 2012, 2013), or the integration of sensory signals over time (Joo et al., 2017). Broadly speaking, skilled reading requires rapid communication among a distributed network of visual, auditory, and language processing systems and an impairment in any one of these systems, or the connections between them, could cause difficulties learning a complex skill like reading (Wandell & Yeatman, 2013).

Our main conclusion is a lack of concordance with either a single deficit or cascading deficit model. Evidence derives from the Healthy Brain Network public dataset (Study 1), which was modestly consistent with a phonological‐core model of dyslexia and but also strongly suggested the need for other predictors to (1) avoid vast overprediction (low specificity) of reading disability in the general population and (2) explain the cases of dyslexia that occur without a clear phonological impairment. Further evidence comes from Study 2, in which several forms of modeling suggested both direct and indirect influences of visual processing on reading skill; a cascading model would predict that parameters from the DDM are useful only insofar as they relate to phonological processing, but mediation analysis and factor analysis were both consistent with the presence of multiple distinct latent variables that combine additively to explain reading skill. As such, our results contradict claims that a single mechanism, either phonological, visual, or non‐sensory, can be considered the “fundamental” or “core” deficit of dyslexia.

The clinical implications of this multifactorial model are an important target for future research. Whether or not different risk profiles predict outcomes for children enrolled in competing intervention programs is an empirical question that cannot be readily inferred from correlational data. For example, in a previous intervention study we demonstrated that individual differences in visual motion sensitivity have no prognostic value for predicting a child's response to intervention (at least for the intervention approach we employed, Joo et al., 2017).

Moving forward, we propose an additive risk factor mode of dyslexia in which multiple dimensions of sensory, cognitive, and linguistic processes contribute distinct risk for reading difficulties. Our results are agnostic to whether poor performance on any given task indicates deficits in the specific targeted function (e.g., motion processing) or indexes processing capacities of a broader system (e.g., constructing a high‐fidelity representation of a noisy visual signal). There are also likely to be dimensions that we have not explored here, as there is growing evidence for a unique role of oral language and vocabulary skills in reading development (Catts et al., 2017; Snowling, 2008; Snowling & Melby‐Lervåg, 2016).

In sum, an additive model outperforms cascading deficit models or models that only consider measures of phonological processing without considering the role of sensory processing. Rather than continuing to seek a single underlying cause of dyslexia, the field should systematically build toward a more complete model of the factors that add risk (or protection) for reading difficulties. Our data and model necessitate a shift toward theories that explain skilled and disabled reading as emerging from a high‐dimensional space determined by several distinct processing systems.

## CONFLICT OF INTEREST

We have no conflict of interest to report.

## Data Availability

The de‐identified datasets reported and analyzed in this manuscript, as well as scripts for stimulus presentation, data analysis, and figure generation are available at: https://github.com/YeatmanLab/Parametric_public.

## References

[desc13039-bib-0001] Ahissar, M. (2007). Dyslexia and the anchoring‐deficit hypothesis. Trends in Cognitive Sciences, 11(11), 458–465. 10.1016/j.tics.2007.08.015 17983834

[desc13039-bib-0002] American Psychiatric Association . (2013). DSM‐5 diagnostic classification. In Diagnostic and statistical manual of mental disorders. 10.1176/appi.books.9780890425596.x00diagnosticclassification

[desc13039-bib-0003] Amitay, S. , Ben‐Yehudah, G. , Banai, K. , & Ahissar, M. (2002). Disabled readers suffer from visual and auditory impairments but not from a specific magnocellular deficit. Brain, 125(Pt. 10), 2272–2285. 10.1093/brain/awf231 12244084

[desc13039-bib-0004] Banai, K. , & Ahissar, M. (2004). Poor frequency discrimination probes dyslexics with particularly impaired working memory. Audiology and Neuro‐Otology, 9(6), 328–340. 10.1159/000081282 15467286

[desc13039-bib-0005] Banai, K. , & Ahissar, M. (2006). Auditory processing deficits in dyslexia: Task or stimulus related? Cerebral Cortex, 16(12), 1718–1728. 10.1093/cercor/bhj107 16407480

[desc13039-bib-0006] Bates, D. , Sarkar, D. , & Matrix, L. (2007). The lme4 package. 10.18637/jss.v067.i01

[desc13039-bib-0007] Bosse, M. L. , Tainturier, M. J. , & Valdois, S. (2007). Developmental dyslexia: The visual attention span deficit hypothesis. Cognition, 104(2), 198–230. 10.1016/j.cognition.2006.05.009 16859667

[desc13039-bib-0008] Casini, L. , Pech‐Georgel, C. , & Ziegler, J. C. (2018). It’s about time: revisiting temporal processing deficits in dyslexia. Developmental Science, 21(2), e12530. 10.1111/desc.12530 28239921

[desc13039-bib-0009] Catts, H. W. , McIlraith, A. , Bridges, M. S. , & Nielsen, D. C. (2017). Viewing a phonological deficit within a multifactorial model of dyslexia. Reading and Writing, 30(3), 613–629. 10.1007/s11145-016-9692-2

[desc13039-bib-0010] Colling, L. J. , Noble, H. L. , & Goswami, U. (2017). Neural entrainment and sensorimotor synchronization to the beat in children with developmental dyslexia: An EEG study. Frontiers in Neuroscience, 11, 360. 10.3389/fnins.2017.00360 28747870 PMC5506338

[desc13039-bib-0011] Costello, A. B. , & Osborne, J. W. (2005). Best practices in exploratory factor analysis: Four recommendtions for getting the most from your analysis. Practical Assessment, Research & Evaluation, 10, 7.

[desc13039-bib-0012] Ferguson, E. , & Cox, T. (1993). Exploratory factor analysis: A users’guide. International Journal of Selection and Assessment, 1, 84–94. 10.1111/j.1468-2389.1993.tb00092.x

[desc13039-bib-0013] Filzmoser, P. (2004). A multivariate outlier detection method. Seventh international conference on computer data analysis and modeling (pp. 18–22), Belarusian State University, Minsk, Belarus.

[desc13039-bib-0014] Frey, A. , François, C. , Chobert, J. , Besson, M. , & Ziegler, J. C. (2019). Behavioral and electrophysiological investigation of speech perception deficits in silence, noise and envelope conditions in developmental dyslexia. Neuropsychologia, 130, 3–12. 10.1016/j.neuropsychologia.2018.07.033 30075216

[desc13039-bib-0015] Frey, A. , François, C. , Chobert, J. , Velay, J. L. , Habib, M. , & Besson, M. (2019). Music training positively influences the preattentive perception of voice onset time in children with dyslexia: A longitudinal study. Brain Sciences, 9(4), 91. 10.3390/brainsci9040091 PMC6523730 31010099

[desc13039-bib-0016] Friedman, J. , Hastie, T. , & Tibshirani, R. (2010). Regularization paths for generalized linear models via coordinate descent. Journal of Statistical Software, 33(1). 10.18637/jss.v033.i01 PMC2929880 20808728

[desc13039-bib-0017] Gabay, Y. , Thiessen, E. D. , & Holt, L. L. (2015). Impaired statistical learning in developmental dyslexia. Journal of Speech Language and Hearing Research, 58(3), 934. 10.1044/2015_JSLHR-L-14-0324 PMC4490081 25860795

[desc13039-bib-0018] Gold, J. I. , & Shadlen, M. N. (2007). The neural basis of decision making. Annual Review of Neuroscience, 30, 535–574. 10.1146/annurev.neuro.29.051605.113038 17600525

[desc13039-bib-0019] Gori, S. , Seitz, A. R. , Ronconi, L. , Franceschini, S. , & Facoetti, A. (2016). Multiple causal links between magnocellular‐dorsal pathway deficit and developmental dyslexia. Cerebral Cortex, 26(11), 4356–4369. 10.1093/cercor/bhv206 26400914 PMC6317503

[desc13039-bib-0020] Goswami, U. (2015). Sensory theories of developmental dyslexia: three challenges for research. Nature Reviews Neuroscience, 16(1), 43–54. 10.1038/nrn3836 25370786

[desc13039-bib-0021] Haft, S. L. , Myers, C. A. , & Hoeft, F. (2016). Socio‐emotional and cognitive resilience in children with reading disabilities. Current Opinion in Behavioral Sciences, 10, 133–141. 10.1016/j.cobeha.2016.06.005 27747263 PMC5058360

[desc13039-bib-0022] Hämäläinen, J. A. , Salminen, H. K. , & Leppänen, P. H. T. (2013). Basic auditory processing deficits in dyslexia. Journal of Learning Disabilities, 46(5), 413–427. 10.1177/0022219411436213 22323280

[desc13039-bib-0023] Hayton, J. C. , Allen, D. G. , & Scarpello, V. (2004). Factor retention decisions in exploratory factor analysis: A tutorial on parallel analysis. Organizational Research Methods, 7(2), 191–205. 10.1177/1094428104263675

[desc13039-bib-0024] Ho, C. S. H. , Chan, D. W. O. , Tsang, S. M. , & Lee, S. H. (2002). The cognitive profile and multiple‐deficit hypothesis in Chinese developmental dyslexia. Developmental Psychology, 38(4), 543. 10.1037/0012-1649.38.4.543 12090484

[desc13039-bib-0025] Huang‐Pollock, C. , Ratcliff, R. , McKoon, G. , Shapiro, Z. , Weigard, A. , & Galloway‐Long, H. (2017). Using the diffusion model to explain cognitive deficits in attention deficit hyperactivity disorder. Journal of Abnormal Child Psychology, 45(1), 57–68. 10.1007/s10802-016-0151-y 27030470 PMC5045756

[desc13039-bib-0026] Hulme, C. , Nash, H. M. , Gooch, D. , Lervåg, A. , & Snowling, M. J. (2015). The foundations of literacy development in children at familial risk of dyslexia. Psychological Science, 26(12), 1877–1886. 10.1177/0956797615603702 26525072 PMC4676358

[desc13039-bib-0027] Jaffe‐Dax, S. , Frenkel, O. , & Ahissar, M. (2017). Dyslexics’ faster decay of implicit memory for sounds and words is manifested in their shorter neural adaptation. Elife, 6, 1–19. 10.7554/eLife.20557.001 PMC5279949 28115055

[desc13039-bib-0028] Joo, S. J. , Donnelly, P. M. , & Yeatman, J. D. (2017). The causal relationship between dyslexia and motion perception reconsidered. Scientific Reports, 7(1). 10.1038/s41598-017-04471-5 PMC5482857 28646168

[desc13039-bib-0029] Kline, R. (2013). Exploratory and confirmatory factor analysis. In Y. Petscher & C. Schatsschneider (Eds.), Applied quantitative analysis in education and the social sciences. Routledge. 10.4324/9780203108550

[desc13039-bib-0030] Krause, M. B. (2015). Pay attention!: sluggish multisensory attentional shifting as a core deficit in developmental dyslexia. Dyslexia, 21(4), 285–303. 10.1002/dys.1505 26338085

[desc13039-bib-0031] Lieder, I. , Adam, V. , Frenkel, O. , Jaffe‐Dax, S. , Sahani, M. , & Ahissar, M. (2019). Perceptual bias reveals slow‐updating in autism and fast‐forgetting in dyslexia. Nature Neuroscience, 22(2), 256–264. 10.1038/s41593-018-0308-9 30643299

[desc13039-bib-0032] Lobier, M. , & Valdois, S. (2015). Visual attention deficits in developmental dyslexia cannot be ascribed solely to poor reading experience. Nature Reviews Neuroscience, 16(4), 1. 10.1038/nrn3836-c1 25790867

[desc13039-bib-0033] Lobier, M. , Zoubrinetzky, R. , & Valdois, S. (2012). The visual attention span deficit in dyslexia is visual and not verbal. Cortex, 48(6), 768–773. 10.1016/j.cortex.2011.09.003 21982580

[desc13039-bib-0034] Menghini, D. , Carlesimo, G. A. , Marotta, L. , Finzi, A. , & Vicari, S. (2010). Developmental dyslexia and explicit long‐term memory. Dyslexia, 16(3), 213–225. 10.1002/dys.410 20680992

[desc13039-bib-0035] Miller, G. A. , & Chapman, J. P. (2001). Misunderstanding analysis of covariance. Journal of Abnormal Psychology, 110(1), 40–48. 10.1037/0021-843X.110.1.40 11261398

[desc13039-bib-0036] Mitchell, J.‐J. (2001). Comprehensive test of phonological processing. Assessment for Effective Intervention. 10.1177/073724770102600305

[desc13039-bib-0037] Muter, V. , & Snowling, M. J. (2009). Children at familial risk of dyslexia: Practical implications from an at‐risk study. Child and Adolescent Mental Health, 14(1), 37–41. 10.1111/j.1475-3588.2007.00480.x

[desc13039-bib-0038] Nicolson, R. I. , & Fawcett, A. J. (2018). Procedural learning, dyslexia and delayed neural commitment. In T. Lachmann & T. Weis (Eds.), Reading and dyslexia (pp. 235–269). Springer.

[desc13039-bib-0039] Noordenbos, M. W. , & Serniclaes, W. (2015). The categorical perception deficit in dyslexia: A meta‐analysis. Scientific Studies of Reading, 19(5), 340–359. 10.1080/10888438.2015.1052455

[desc13039-bib-0040] O’Brien, G. E. , McCloy, D. R. , Kubota, E. C. , & Yeatman, J. D. (2018). Reading ability and phoneme categorization. Scientific Reports, 8(1). 10.1038/s41598-018-34823-8 PMC6237901 30442952

[desc13039-bib-0041] Palmer, J. , Huk, A. C. , & Shadlen, M. N. (2005). The effect of stimulus strength on the speed and accuracy of a perceptual decision. Journal of Vision, 5(5), 1. 10.1167/5.5.1 16097871

[desc13039-bib-0042] Pennington, B. F. (2006). From single to multiple deficit models of developmental disorders. Cognition, 101(2), 385–413. 10.1016/j.cognition.2006.04.008 16844106

[desc13039-bib-0043] Pennington, B. F. , Santerre‐lemmon, L. , Rosenberg, J. , Macdonald, B. , Boada, R. , Friend, A. , Leopold, D. R. , Samuelsson, S. , Byrne, B. , Willcutt, E. G. , & Olson, R. K. (2012). Individual prediction of dyslexia by single vs. multiple deficit models. Journal of Abnormal Psychology, 121(1), 212–224. 10.1037/a0025823.Individual 22022952 PMC3270218

[desc13039-bib-0044] Perrachione, T. K. , Del Tufo, S. N. , Winter, R. , Murtagh, J. , Cyr, A. , Chang, P. , Halverson, K. , Ghosh, S. S. , Christodoulou, J. A. , & Gabrieli, J. D. E. (2016). Dysfunction of rapid neural adaptation in dyslexia. Neuron, 92(6), 1383–1397. 10.1016/j.neuron.2016.11.020 28009278 PMC5226639

[desc13039-bib-0045] Peterson, R. L. , & Pennington, B. F. (2015). Developmental dyslexia. Annual Review of Clinical Psychology, 11, 283–307. 10.1016/S0140-6736(12)60198-6 25594880

[desc13039-bib-0046] Ramus, F. , & Ahissar, M. (2012). Developmental dyslexia: The difficulties of interpreting poor performance, and the importance of normal performance. Cognitive Neuropsychology, 29(1–2), 104–122. 10.1080/02643294.2012.677420 22559749

[desc13039-bib-0047] Ramus, F. , Rosen, S. , Dakin, S. C. , Day, B. L. , Castellote, J. M. , White, S. , & Frith, U. (2003). Theories of developmental dyslexia: Insights from a multiple case study of dyslexic adults. Brain, 126(4), 841–865. 10.1093/brain/awg076 12615643

[desc13039-bib-0048] Ratcliff, R. , Love, J. , Thompson, C. A. , & Opfer, J. E. (2012). Children are not like older adults: A diffusion model analysis of developmental changes in speeded responses. Child Development, 83(1), 367–381. 10.1111/j.1467-8624.2011.01683.x 22188547 PMC3267006

[desc13039-bib-0049] Ratcliff, R. , & McKoon, G. (2008). The diffusion decision model: Theory and data for two‐choice decision tasks. Neural Computation, 20(1), 873–922. 10.1016/j.ejphar.2004.07.002 18085991 PMC2474742

[desc13039-bib-0050] Ratcliff, R. , McKoon, G. , & Gomez, P. (2004). A diffusion model account of the lexical decision task. Psychological Review, 111(1), 159–182. 10.1037/0033-295X.111.1.159 14756592 PMC1403837

[desc13039-bib-0051] Ratcliff, R. , Thapar, A. , & McKoon, G. (2004). A diffusion model analysis of the effects of aging on recognition memory. Journal of Memory and Language. 10.1016/j.jml.2003.11.002

[desc13039-bib-0052] Saksida, A. , Iannuzzi, S. , Bogliotti, C. , Chaix, Y. , Démonet, J.‐F. , Bricout, L. , Billard, C. , Nguyen‐Morel, M.‐A. , Le Heuzey, M.‐F. , Soares‐Boucaud, I. , George, F. , Ziegler, J. C. , & Ramus, F. (2016). Phonological skills, visual attention span, and visual stress in developmental dyslexia. Developmental Psychology, 52(10), 1503–1516. 10.1037/dev0000184 27690491

[desc13039-bib-0053] Sandon, F. , & Wald, A. (1947). Sequential analysis. The Mathematical Gazette, 33(303), 66. 10.2307/3608454

[desc13039-bib-0054] Schrank, F. A. , McGrew, K. S. , Mather, N. , Wendling, B. J. , & LaForte, E. M. (2014). Woodcock‐Johnson IV tests of achievement. Riverside Publishing Company.

[desc13039-bib-0055] Shadlen, M. N. , Hanks, T. D. , Churchland, A. K. , Kiani, R. , & Yang, T. (2013). The Speed and Accuracy of a Simple Perceptual Decision: A Mathematical Primer. In D. Kenji , I. Shin , P. Alexandre & R. P. N. Rajesh (Eds.), Bayesian brain (pp. 207–236). MIT Press. 10.7551/mitpress/9780262042383.003.0010

[desc13039-bib-0056] Shadlen, M. N. , & Newsome, W. T. (2001). Neural basis of a perceptual decision in the parietal cortex (area LIP) of the rhesus monkey. Journal of Neurophysiology, 86(4), 1916–1936. Retrieved from http://www.ncbi.nlm.nih.gov/pubmed/11600651 11600651 10.1152/jn.2001.86.4.1916

[desc13039-bib-0057] Shaywitz, S. E. , Escobar, M. D. , Shaywitz, B. A. , Fletcher, J. M. , & Makuch, R. (1992). Evidence that dyslexia may represent the lower tail of a normal distribution of reading ability. New England Journal of Medicine, 326(3), 145–150. 10.1056/NEJM199201163260301 1727544

[desc13039-bib-0058] Smith, P. L. , & Ratcliff, R. (2004). Psychology and neurobiology of simple decisions. Trends in Neurosciences, 27(3), 161–168. 10.1016/j.tins.2004.01.006 15036882

[desc13039-bib-0059] Snowling, M. J. (2008). Specific disorders and broader phenotypes: The case of dyslexia. Quarterly Journal of Experimental Psychology. 10.1080/17470210701508830 18038345

[desc13039-bib-0060] Snowling, M. J. , Lervåg, A. , Nash, H. M. , & Hulme, C. (2019). Longitudinal relationships between speech perception, phonological skills and reading in children at high‐risk of dyslexia. Developmental Science. 10.1111/desc.12723 PMC6492008 30207641

[desc13039-bib-0061] Snowling, M. J. , & Melby‐Lervåg, M. (2016). Oral language deficits in familial dyslexia: A meta‐analysis and review. Psychological Bulletin. 10.1037/bul0000037 PMC4824243 26727308

[desc13039-bib-0062] Sperling, A. J. , Lu, Z.‐L. , Manis, F. R. , & Seidenberg, M. S. (2005). Deficits in perceptual noise exclusion in developmental dyslexia. Nature Neuroscience, 8(7), 862–863. 10.1038/nn1474 15924138

[desc13039-bib-0063] Sperling, A. J. , Lu, Z. L. , Manis, F. R. , & Seidenberg, M. S. (2006). Motion‐perception deficits and reading impairment: It’s the noise, not the motion. Psychological Science, 17(12), 1047–1053. 10.1111/j.1467-9280.2006.01825.x 17201786

[desc13039-bib-0064] Stanovich, K. E. (1988). Explaining the differences between the dyslexic and the garden‐variety poor reader. Journal of Learning Disabilities, 21(10), 590–604. 10.1177/002221948802101003 2465364

[desc13039-bib-0065] Stein, J. (2001). The magnocellular theory of developmental dyslexia. Dyslexia, 7(1), 12–36. 10.1002/dys.186 11305228

[desc13039-bib-0066] Stein, J. (2019). The current status of the magnocellular theory of developmental dyslexia. Neuropsychologia, 130, 66–77. 10.1016/j.neuropsychologia.2018.03.022 29588226

[desc13039-bib-0067] Stein, J. , & Talcott, J. (1999). Impaired neuronal timing in developmental dyslexia—The magnocellular hypothesis. Dyslexia, 5(2), 59–77. 10.1002/(SICI)1099-0909(199906)5:2<59:AID-DYS134>3.0.CO;2-F

[desc13039-bib-0068] Stein, J. , & Walsh, V. (1997). To see but not to read; the magnocellular theory of dyslexia. Trends in Neurosciences, 20(4), 147–152. Retrieved from http://www.ncbi.nlm.nih.gov/pubmed/9106353 9106353 10.1016/s0166-2236(96)01005-3

[desc13039-bib-0069] Stuart, G. W. , McAnally, K. I. , McKay, A. , Johnston, M. , & Castles, A. (2006). A test of the magnocellular deficit theory of dyslexia in an adult sample. Cognitive Neuropsychology, 23(8), 1215–1229. 10.1080/02643290600814624 21049375

[desc13039-bib-0070] Talcott, J. B. , Hansen, P. C. , Assoku, E. L. , & Stein, J. F. (2000). Visual motion sensitivity in dyslexia: Evidence for temporal and energy integration deficits. Neuropsychologia, 38(7), 935–943. 10.1016/S0028-3932(00)00020-8 10775704

[desc13039-bib-0071] Talcott, J. B. , Witton, C. , Hebb, G. S. , Stoodley, C. J. , Westwood, E. A. , France, S. J. , Hansen, P. C. , & Stein, J. F. (2002). On the relationship between dynamic visual and auditory processing and literacy skills; results from a large primary‐school study. Dyslexia, 8(4), 204–225. 10.1002/dys.224 12455851

[desc13039-bib-0072] Talcott, J. B. , Witton, C. , Mclean, M. F. , Hansen, P. C. , Rees, A. , Green, G. G. R. , & Stein, J. F. (2000). Dynamic sensory sensitivity and children’s word decoding skills. Proceedings of the National Academy of Sciences of the United States of America, 97(6), 2952–2957. 10.1073/pnas.040546597 10688885 PMC16036

[desc13039-bib-0073] Torgesen, J. K. , Wagner, R. , & Rashotte, C. (2011). TOWRE 2: Test of word reading efficiency. Test of word reading efficiency. In Pearson Clinical Assessment. 10.1007/BF02245613

[desc13039-bib-0074] Van Ingelghem, M. , Van Wieringen, A. , Wouters, J. , Vandenbussche, E. , Onghena, P. , & Ghesquière, P. (2001). Psychophysical evidence for a general temporal processing deficit in children with dyslexia. NeuroReport, 12(16), 3603–3607. 10.1097/00001756-200111160-00046 11733720

[desc13039-bib-0075] Van Zandt, T. (2011). How to fit a response time distribution. Psychonomic Bulletin & Review, 7(3), 424–465. 10.3758/bf03214357 11082851

[desc13039-bib-0076] Vandermosten, M. , Wouters, J. , Ghesquière, P. , & Golestani, N. (2018). Statistical learning of speech sounds in dyslexic and typical reading children. Scientific Studies of Reading, 23(1), 116–127. 10.1080/10888438.2018.1473404

[desc13039-bib-0077] Vidyasagar, T. R. (2019). Visual attention and neural oscillations in reading and dyslexia: Are they possible targets for remediation? Neuropsychologia, 130, 59–65. 10.1016/j.neuropsychologia.2019.02.009 30794841

[desc13039-bib-0078] Wandell, B. A. , & Yeatman, J. D. (2013). Biological development of reading circuits. Current Opinion in Neurobiology, 23(2), 261–268. 10.1016/j.conb.2012.12.005 23312307 PMC3622751

[desc13039-bib-0079] Ward, J. H. (1963). Hierarchical grouping to optimize an objective function. Journal of the American Statistical Association, 58, 236–244. 10.1080/01621459.1963.10500845

[desc13039-bib-0080] Wechsler, D. (2011). Wechsler Abbreviated Scale of Intelligence‐second edition, manual. Pearson.

[desc13039-bib-0081] White, S. , Milne, E. , Rosen, S. , Hansen, P. , Swettenham, J. , Frith, U. , & Ramus, F. (2006). The role of sensorimotor impairments in dyslexia: A multiple case study of dyslexic children. Developmental Science. 10.1111/j.1467-7687.2006.00483.x 16669791

[desc13039-bib-0082] Wiecki, T. V. , Sofer, I. , & Frank, M. J. (2013). HDDM: hierarchical Bayesian estimation of the drift‐diffusion model in python. Frontiers in Neuroinformatics. 10.3389/fninf.2013.00014 PMC3731670 23935581

[desc13039-bib-0083] Witton, C. , Talcott, J. B. , Hansen, P. C. , Richardson, A. J. , Griffiths, T. D. , Rees, A. , Stein, J. F. , & Green, G. (1998). Sensitivity to dynamic auditory and visual stimuli predicts nonword reading ability in both dyslexic and normal readers. Current Biology, 8(14), 791–797. 10.1016/S0960-9822(98)70320-3 9663387

[desc13039-bib-0084] Wolf, M. , & Bowers, P. G. (1999). The double‐deficit hypothesis for the developmental dyslexias. Journal of Educational Psychology. 10.1037/0022-0663.91.3.415

[desc13039-bib-0085] Wolf, M. , & Bowers, P. G. (2000). Naming‐speed processes and developmental reading disabilities: An introduction to the special issue on the double‐deficit hypothesis. Journal of Learning Disabilities. 10.1177/002221940003300404 15493094

[desc13039-bib-0086] Yeatman, J. D. , Dougherty, R. F. , Ben‐Shachar, M. , & Wandell, B. A. (2012). Development of white matter and reading skills. Proceedings of the National Academy of Sciences of the United States of America, 109(44), E3045–E3053. 10.1073/pnas.1206792109 23045658 PMC3497768

[desc13039-bib-0087] Yeatman, J. D. , Rauschecker, A. M. A. M. , & Wandell, B. A. (2013). Anatomy of the visual word form area: Adjacent cortical circuits and long‐range white matter connections. Brain and Language, 125(2), 146–155. 10.1016/j.bandl.2012.04.010 22632810 PMC3432298

[desc13039-bib-0088] Zeguers, M. H. T. , Snellings, P. , Tijms, J. , Weeda, W. D. , Tamboer, P. , Bexkens, A. , & Huizenga, H. M. (2011). Specifying theories of developmental dyslexia: A diffusion model analysis of word recognition. Developmental Science, 14(6), 1340–1354. 10.1111/j.1467-7687.2011.01091.x 22010894

[desc13039-bib-0089] Ziegler, J. C. (2008). Better to lose the anchor than the whole ship. Trends in Cognitive Sciences, 12(7), 244–245. 10.1016/j.tics.2008.04.001 18515174

[desc13039-bib-0090] Zoubrinetzky, R. , Bielle, F. , & Valdois, S. (2014). New insights on developmental dyslexia subtypes: Heterogeneity of mixed reading profiles. PLoS One, 9(6). 10.1371/journal.pone.0099337 PMC4053380 24918441

