# Supplementary material for: Bridging sensory and language theories of dyslexia: Toward a multifactorial model
Source: Dev Sci. 2020 Oct 19;24(3):e13039. doi: 10.1111/desc.13039 (PMC8244000; doi:10.1111/desc.13039)
Supplement: Supplementary file 1 [file DESC-24-e13039-s001.docx]

Supplementary Methods

**Psychophysics stimuli**

Stimuli for the motion discrimination experiment were created using MATLAB (The Mathworks Corporation, Natick, MA, USA) in conjunction with the Psychophysics Toolbox. Stimuli were displayed on a LG liquid crystal display (1,920 × 1,080 resolution, 120 Hz refresh rate, subtending 51° horizontally). The subjects’ response was collected using keypresses. The viewing distance was 56 cm. We used random-dot motion stimuli (150 dots) that were displayed in a circular aperture (14° in diameter) centered around the fixation mark (1°) at the center of display. Light (271 cd/m^2^) and dark (0 cd/m^2^) dots (dot size = 0.15°) moved at the speed of 8°/s on a gray background (135 cd/m^2^). Each dot was assigned a random lifetime from a uniform distribution between 0 and 200 ms (24 video frames). When a dot’s lifetime expired, it was randomly re-positioned within the aperture and assigned the maximum lifetime (200 ms). Motion coherence was defined as the percentage of dots moving together in the same direction compared to dots moving in random directions. The stimuli were equivalent to those used in Joo *et al*.(Joo, Donnelly, & Yeatman, 2017) except (a) with fixed coherence levels, and (b) stimuli remained on the screen until the subject indicated a decision with a button press (as opposed to fixed duration).

**Drift diffusion model**

Fundamentally, the DDM tries to maximize the likelihood of observing a distribution of reaction times according to the probability density function


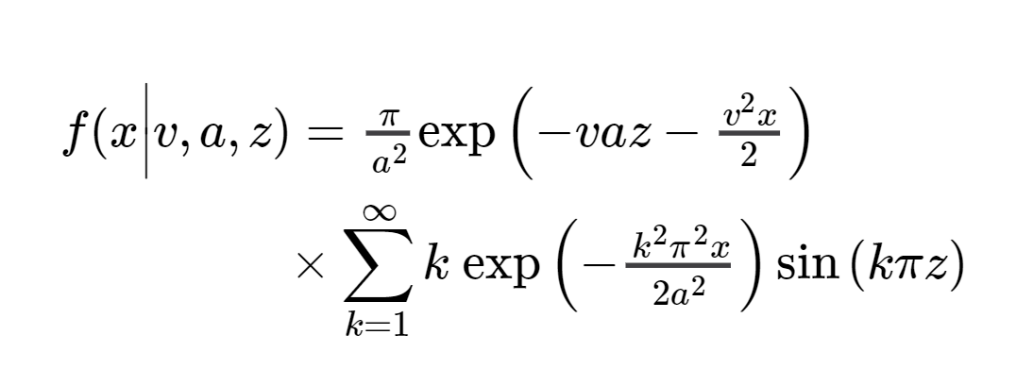


where *x* is reaction time, *v* is drift rate (the average rate at which evidence is accumulated for a decision), *a* is the distance between the two decision boundaries, and *z* is a bias term that allows for an observer to prefer one alternative to the other(Wald, 1947). The parameter *v* is allowed to vary with stimulus level. Additionally, the DDM fits a parameter *t*, which corresponds to the non-decision time- in other words, the time taken by all sensory and motor processes besides accumulating evidence for a decision, such as planning and executing a motor response and converting incoming sensory inputs to units of evidence.

The above equation predicts perfectly symmetric distributions of correct and error response times, and so the DDM model has been extended to include three free parameters that allow it to approximate more realistic distributions. The first of these parameters is *sz*, the trial-to-trial variability in the drift process starting point (centered around the halfway-point between the two decision bounds), which allows the model to predict fast errors. The other parameters are *sv*, the trial-to-trial variability in average drift rate, and *st*, the trial-to-trial variability in residual time *t*. When the DDM is fit with these three additional parameters, it is referred to as the *full* DDM.

The full DDM was fit using the Hierarchical Drift Diffusion Model toolkit for Python (Wiecki et al., 2013). Parameter reliability estimates are provided in the supplement (Table S3). The DDM was fit to each individual’s behavioral responses and reaction times using the Maximum Likelihood fitting method (as recommended by Van Zandt (2011)). The optimization scheme was attempted five times per individual and parameter estimates from the best run were saved. In all cases, the optimization scheme terminated successfully. As recommended by the makers of the HDDM package (Wiecki et al., 2013), the DDM was fit with a mixture model that allowed up to 5% of responses to be assigned to a uniform “lapse” distribution. This reduces bias in drift rate estimates due to occasional lapses. Because this mixture component was included, we employed only a coarse screen for outlier detection before DDM fitting: responses occurring before 200 ms (before a typical behavioral response can be executed) and after 10s (after the stimulus had concluded) were excluded. Although we excluded two participants with >15% data loss, the average participant in the remaining sample had 98.1% usable data.

## **Outlier detection**

To determine the presence of highly unusual model fits, we computed the Mahalanobis distance for each individual with respect to the 9 parameters estimated by the DDM. The Mahalanobis distance for multiple dimensions follows a chi-squared distribution, and so we use this measure to detect outliers (Filzmoser, 2004). Specifically, individuals with a Mahalanobis distance corresponding to values beyond the p < 0.001 threshold were deemed to be outliers. Two such individuals were detected; both had been fit with extremely high *a* values (*a* = 8.17 and *a* = 5.60). One of these individuals had a composite reading score in the Dyslexic range, whereas the other would have been above our cutoff. These two points were excluded from further analysis as we have cause to doubt the quality of their DDM parameter estimates, but their results are included with the full dataset online.

## **Stepwise model selection procedure**

In our analyses of the relationships between various parameter estimates from the DDM and reading skill, we employed a stepwise model selection procedure. In all cases, we considered three covariates: age, nonverbal-IQ, and the presence of an ADHD diagnosis. While age and ADHD diagnosis did not significantly correlate with reading skill in our sample (Tables S1 and S2), nonverbal IQ and reading skill were significantly correlated. There is some philosophical debate about the validity of “controlling for” attributes of a diagnosis that share a meaningful covariance in nature (Miller & Chapman, 2001): because reading skill and IQ are known to be correlated in the population, and because an IQ-discrepancy is largely unsupported for diagnosing and treating dyslexia, regressing out variance in reading skill associated with IQ may not always be desirable. Opinions about the separability of IQ and reading skill likely vary by research group. Therefore, although we do include IQ as a covariate in the following analyses, the full dataset and analysis pipeline are made available online so interested readers can assess results with or without controlling for IQ.

Each model selection procedure began with a fully specified model of reading score as a function of the parameter(s) of interest plus the three covariates. Except where specified, reading score is always treated as the dependent variable. Fitting was performed with the base R lm() function, except where mixed model usage is noted, in which case the lme4 library was used (Bates, Sarkar, & Matrix, 2007). The contributions of the covariates were first tested using an anova test. A given covariate was retained if the p-value associated with the more complex model was less than 0.1; otherwise, the covariate was dropped. Next, parameters of interest were tested similarly. Throughout the manuscript, wherever model selection is performed we report the selected (“most parsimonious”) model.

Supplementary Materials

| Table S1: Correlations with composite reading score | | | |
| --- | --- | --- | --- |
| Measure | 𝛽 | SE | *P* |
| Age  ADHD diagnosis  Gender | 1.08  -6.91  1.01 | 1.4  4.45  3.78 | 0.443  0.124  0.789 |
| **CTOPP-2** | | | |
| Phonological Awareness  Phonological Memory  Rapid Automatic Naming | 0.65  0.57  0.88 | 0.11  0.1  0.09 | 3.46 x 10^-8^  8.10 x 10^-8^  1.23 x 10^-15^ |
| **TOWRE-2** | | | |
| Pseudoword Decoding  Sight Word Reading | 1.02  0.82 | 0.03  0.03 | < 1 x 10^-15^  < 1 x 10^-15^ |
| **WASI-II** | | | |
| Full-scale IQ  Matrix Reasoning t-score  Vocabulary t-score | 0.75  0.95  0.97 | 0.09  0.16  0.13 | 2.87 x 10^-13^  3.50 x 10^-8^  4.05 x 10^-11^ |
| **Woodcock Johnson** | | | |
| Letter Word Identification  Word Attack | 0.91  0.94 | 0.03  0.05 | < 1 x 10^-15^  < 1 x 10^-15^ |

| Table S2: Group demographic information | | | | | |
| --- | --- | --- | --- | --- | --- |
|  | **Mean** | | **SD** | | *p* |
|  | Control  *n* = 48 | Dyslexic  *n* = 43 | Control | Dyslexic |  |
| Age  ADHD diagnosis  Gender (♂) | 10  4  28 | 9.69  13  25 | 1.38 | 1.15 | 0.245  0.009  0.985 |
| **CTOPP-2** | | | | | |
| Phonological Awareness  Phonological Memory  Rapid Automatic Naming | 98.33  98.58  99.42 | 86.42  84.86  79 | 14.98  17.32  12.45 | 12.72  12.75  9.63 | 9.09 x 10^-5^  3.96 x 10^-5^  1.15 x 10^-13^ |
| **TOWRE-2** | | | | | |
| TOWRE Index  Pseudoword Decoding  Sight Word Efficiency | 106.79  104.6  108.17 | 68.35  71.58  68.47 | 11  11.38  11.66 | 8.01  6.32  11.3 | < 1 x 10^-15^  < 1 x 10^-15^  < 1 x 10^-15^ |
| **WASI-II** | | | | | |
| Full-Scale IQ  Matrix Reasoning t-score  Vocabulary t-score | 115.77  55.33  63.15 | 96.28  46.63  49.14 | 16.02  10.75  10.99 | 9.87  7.28  7.82 | 5.53 x 10^-10^  1.72 x 10^-5^  4.36 x 10^-10^ |
| **Woodcock Johnson** | | | | | |
| Basic Reading Score  Letter Word Identification  Word Attack | 110.27  109.75  109.35 | 77.23  74.37  82.4 | 12.71  11.79  14.89 | 10.45  12.12  10.84 | < 1 x 10^-15^  < 1 x 10^-15^  6.34 x 10^-16^ |

| Table S3: DDM parameter reliability estimates | | |
| --- | --- | --- |
| Parameter | Split-half reliability^1^ | Adjusted reliability^2^ |
| *v_6_*  *v_12_*  *v_24_*  *v_48_*  *a*  *t*  *sv*  *sz*  *st* | 0.093  0.397  0.353  0.512  0.531  0.594  0.141  0.279  0.470 | 0.170  0.568  0.522  0.677  0.693  0.737  0.248  0.436  0.640 |

^1^ Split half reliability is calculated by partitioning each subject’s responses into two by random assignment, estimating the DDM parameters on each half, and measuring the Pearson’s correlation between parameter estimates.

^2^ Adjusted reliability is calculated with the Spearman-Brown prophecy formula. This adjustment is an estimate of the reliability had the estimates been computed on twice as many observations as in split-half reliability.

| Table S4: Selected model of reaction time on the motion discrimination task | | | |
| --- | --- | --- | --- |
|  | 𝛽 | SE | *p* |
| Intercept  Stimulus coherence  Age  Reading skill | 4.101  -0.173  -0.0590  -0.00600 | 0.316  0.00898  0.0214  0.00149 | < 1 x 10^-15^  < 1 x 10^-15^  0.00703  0.000115 |

| Table S5: Selected model of accuracy on the motion discrimination task | | | |
| --- | --- | --- | --- |
|  | 𝛽 | SE | *p* |
| Intercept  Stimulus coherence  Age | 0.218  0.0844  0.0244 | 0.0618  0.00284  0.00608 | 0.000600  < 1 x 10^-15^  0.000109 |

| Table S6: Selected model of the ratio of error-to-correct-response-times  on the motion discrimination task | | | |
| --- | --- | --- | --- |
|  | 𝛽 | SE | *p* |
| Intercept  Reading skill  Age  Nonverbal IQ | 0.555  0.004440  0.08029  -0.007 | 0.3495  0.002235  0.027793  0.004208 | 0.11534  0.04966  0.00472  0.09859 |


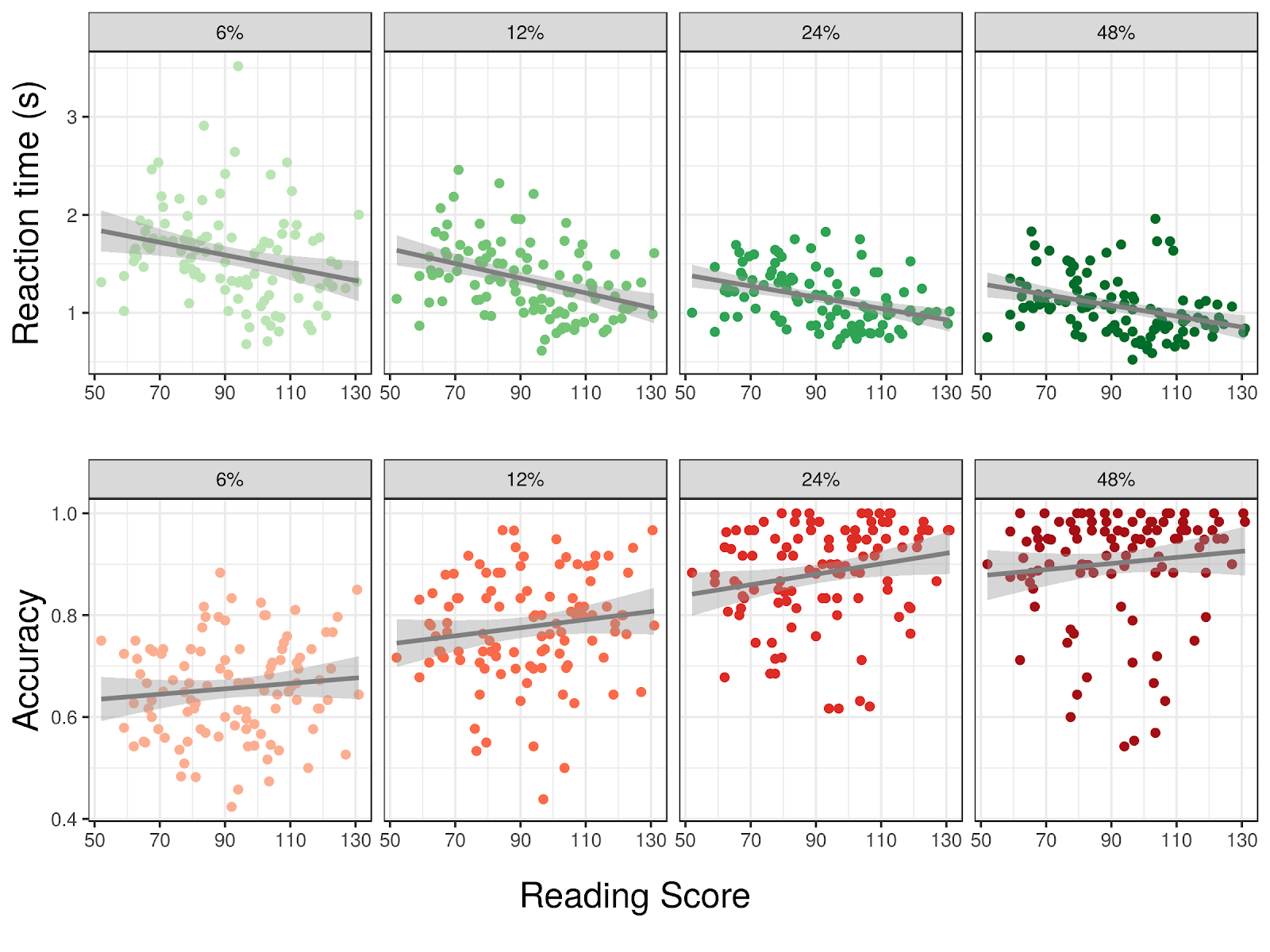


**Figure S1.** *Median reaction times (top row) and accuracy (bottom row) for each individual as a function of reading score. Panels show each stimulus coherence.*


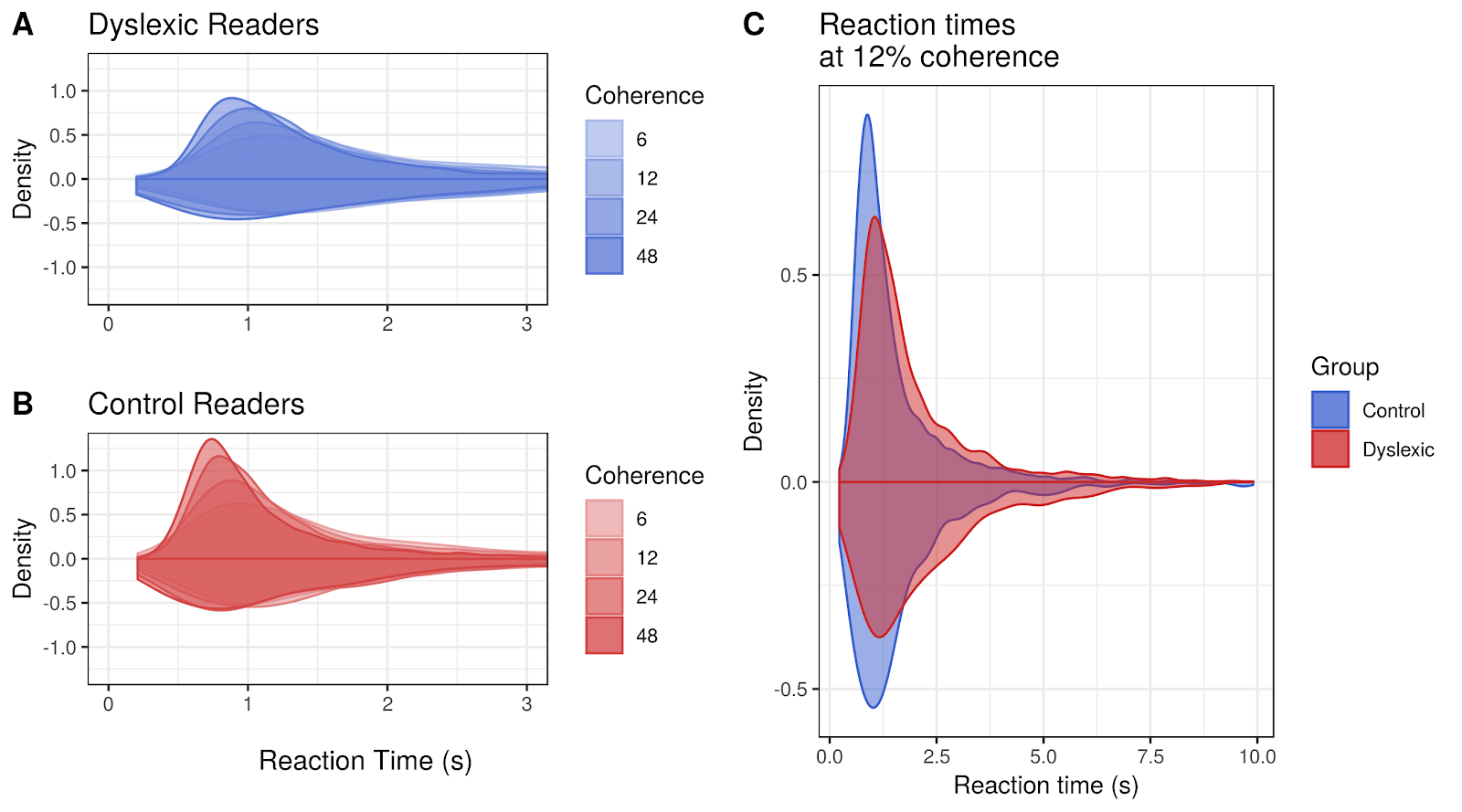


**Figure S2.** *Panels A-B: density plots of reaction times at each coherence level for the Dyslexic and Control groups. On the positive axis, correct response time distributions are shown, and on the negative axis error responses are shown. Plots are truncated at 3 seconds for ease of viewing. Panel C: overlaid density plots of reaction times at 12% coherence for the Dyslexic and Control groups.*

**Supplemental Analysis 1: group level statistics**

*Drift rate:* As in the main manuscript, we used mixed model selection to identify the most parsimonious model of drift rate. Subject was included as a random effect, as our data contained four drift rate estimates per participant (one at each coherence level). The selected model included significant main effects of stimulus coherence and age, plus a significant interaction of stimulus coherence and group (Table S7). The main effect of group was not significant (p = 0.117), but the direction of the relationship was the same as in the model where reading is treated as a continuous variable. Therefore, our results from both models are in qualitative agreement. The fact that reading skill was a significant main effect as a continuous measure but not as a discrete one is likely the result of reduced statistical power: there are 90 subjects in the group analysis, but 104 in the continuous-measure analysis (if all covariates are removed from the model then the main effect of group is significant at p = 0.050).

| Table S7: Selected model of drift rate | | | |
| --- | --- | --- | --- |
|  | 𝛽 | SE | *p* |
| Intercept  Stimulus coherence  Age  Group  Stimulus coherence : Group | 1.630  0.772  0.240  -0.227  -0.133 | 0.0973  0.0265  0.0716  0.143  0.0529 | <2e-16  <2e-16  0.00117  0.117  0.0136 |

We also modeled group as a dependent variable and mean drift rate as a predictor. To model a binary outcome (Dyslexic or Control), we used a generalized linear model with a logit link function. In the selected model, mean drift rate missed the α = 0.10 cutoff for inclusion (*p* = 0.124). Again, the difference between the continuous and group outcome measures is likely due to the loss of statistical power incurred by reducing the number of data points being modeled. Indeed, the estimated magnitude of the effect is similar in both models: in the continuous model, decreasing reading skill by 19 points (1 standard deviation in our sample) is associated with an approximately -0.262 decrease in mean drift rate; in the discrete model, belonging to the Dyslexic group is associated with approximately -0.544 decrease in mean drift rate. The difference in mean reading skill between Dyslexic and Control groups is 36.5 points, or 1.9 standard deviations. Thus, by either model, the change in mean drift rate associated with a 2 standard deviation change in reading skill would be on the order of approximately 0.5, although this main effect was not significant in the group-level model.

*Decision criterion parameters*: The parameter *a*, representing an individual’s threshold of evidence for initiating a decision, was modeled as an independent variable next. Group was modeled as the dependent variable. Generalized mixed model selection dropped the covariate for age but retained nonverbal IQ (𝛽 = -1.102, SE = 0.297, p = 0.0002) and ADHD (𝛽 = 1.761, SE = 0.725, p = 0.0152), as well as *a* (𝛽 = 0.848, SE = 0.415, p = 0.0411).

The parameter *sz*, representing trial-to-trial variability in the drift process starting point, was modeled as a predictor similarly. The main effect of *sz* did not reach the threshold of significance for inclusion in the model (*p* = 0.138).

*Non-decision time parameters:* The selected model of group as a function of residual non-decision time *t* and covariates contained three main effects: *t*, nonverbal IQ and ADHD diagnosis (Table S8).

| Table S8: Selected model of group predicted by residual time *t* | | | |
| --- | --- | --- | --- |
|  | 𝛽 | SE | *p* |
| Intercept  *t*  Nonverbal IQ  ADHD | -3.354  5.484  -1.357  2.324 | 1.063  1.951  0.333  0.808 | 0.00160  0.00500  4.56 x 10^-5^  0.00401 |

Lastly, we considered the trial-to-trial variability in non-decision time *st* as a predictor of group. The selected model contained three predictors: *st*, nonverbal IQ and ADHD diagnosis (Table S9).

| Table S9: Selected model of group predicted by trial-to-trial variability in residual time s*t* | | | |
| --- | --- | --- | --- |
|  | 𝛽 | SE | *P* |
| Intercept  *st*  Nonverbal IQ  ADHD | -2.084  4.041  -1.410  2.311 | 0.584  1.285  0.358  0.800 | 0.000360  0.00167  8.04 x 10^-5^  0.00379 |

We can therefore see that the relationships between estimated DDM parameters and reading skill are qualitatively consistent regardless of whether reading disability is treated as a categorical or continuous variable.


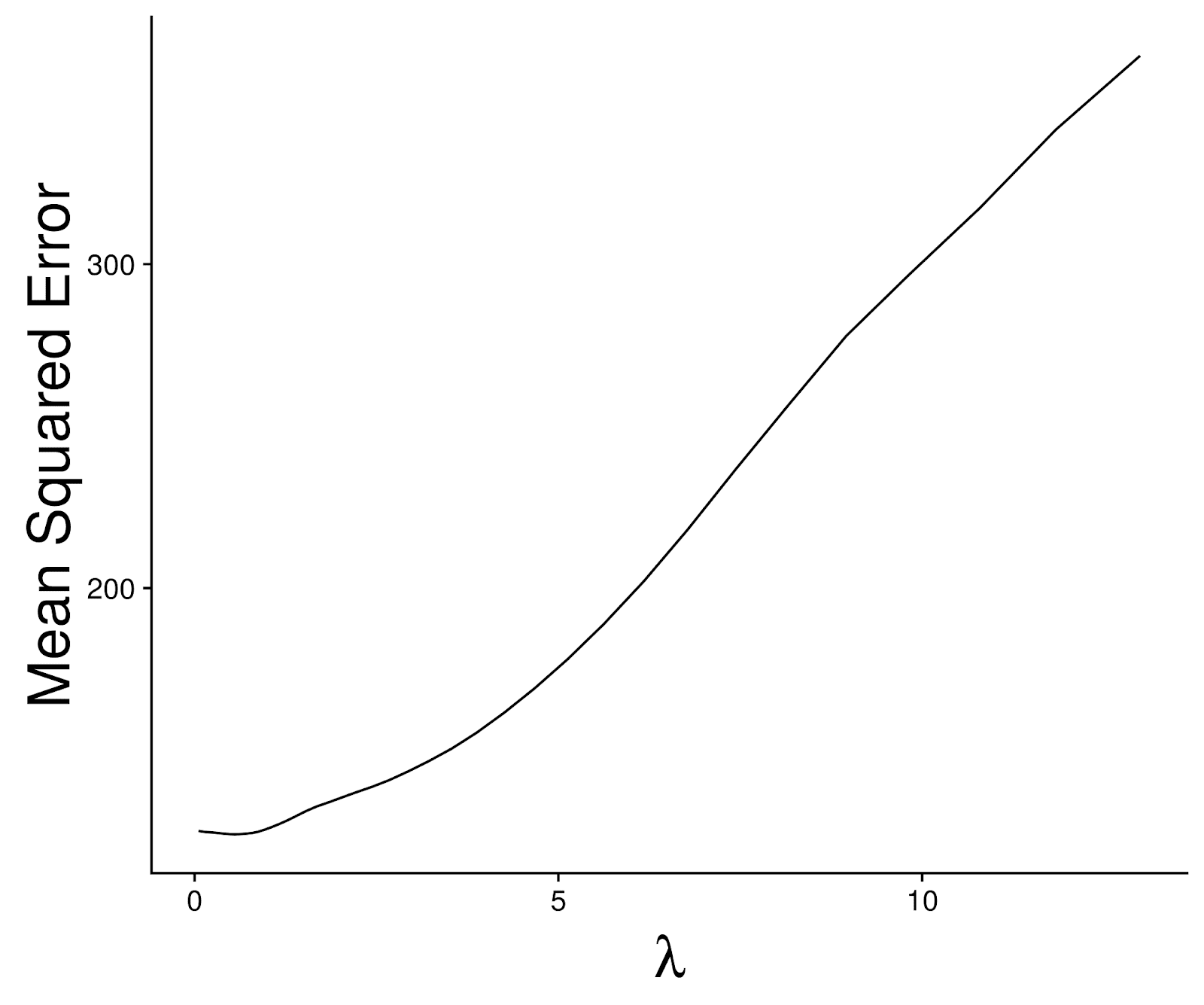


**Figure S3.** *Mean squared error of lasso regression as a function of the regularization parameter λ with 10-fold cross validation.*


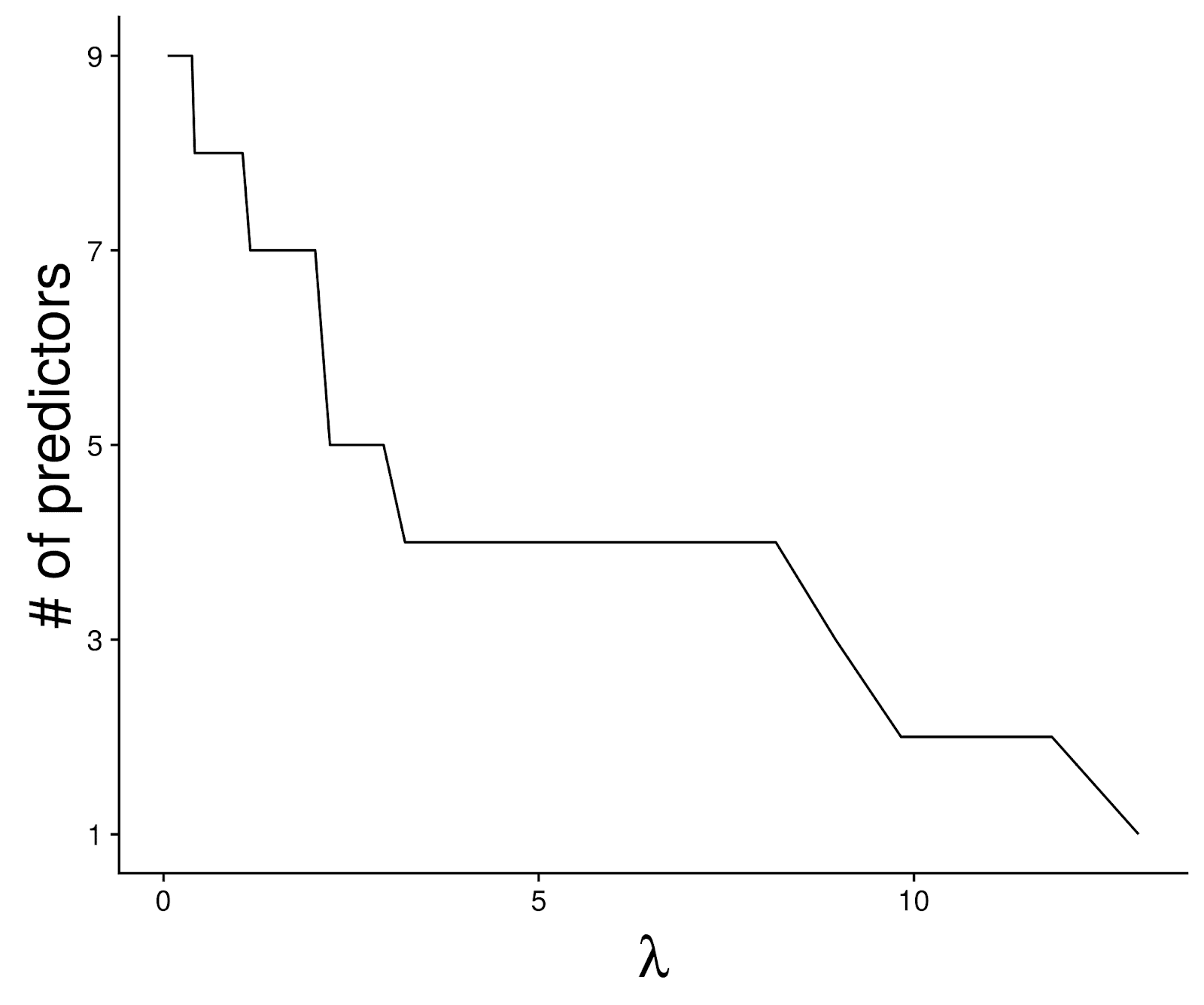


**Figure S4.** *Number of predictors retained by lasso regression as a function of the regularization parameter λ with 10-fold cross validation.*

| Table S10: Lasso model of reading skill | |
| --- | --- |
|  | 𝛽  *(*all measures are scaled prior to lasso regression)* |
| Intercept  *a*  *v_comp_*  *d_comp_*  Nonverbal IQ  CTOPP RAN  CTOPP PA  ADHD diagnosis | -3.277 x 10^-16^  -0.09240  -0.0895  0.118  0.317  0.512  0.169  -0.00221 |

**Supplemental Analysis 2: Mediation analysis**

It has been argued that deficits in sensory processing or decision-making could affect reading skill because they disrupt the typical development of PA. We therefore explored whether this hypothesis is borne out in our data by employing a mediation analysis. Mediation analysis was performed using the “mediation” package for R ^70^. In all mediation models, nonverbal IQ was entered as a covariate. 4000 bootstrap simulations were used to estimate the proportion of mediation of a variable of interest by PA in a linear model of reading skill.

We considered how various composite parameter estimates from the DDM had a relationship with reading skill that was mediated by PA. While *a* was not significantly correlated with PA (and thus not a candidate for mediation), *v_comp_* and *d_comp_* showed modest correlations (*v_comp_* and PA: r = 0.324, *p =* 4.80 x 10^-4^; *d_comp_* and PA: r = 0.182, *p =* 0.0358).

We first tested a model with PA mediating the relationship between *v_comp_* and reading skill and found a significant, partial mediation effect (42.3%, *p =* 0.0052). Similarly, the *d_comp_-*reading skill relationship is partially mediated by PA (22.2% mediation, *p =* 0.0224)), but there was also still a significant direct relationship (𝛽 = 4.293, SE = 1.501, *p =* 0.00516). As such, our results provide some support for the idea that in certain poor readers, low PA could be a consequence of a more fundamental impairment in either sensory or non-sensory mechanisms. However, our data suggest a partial mediation, indicating that many individuals would not be well described by this cascading model and that there are also direct links between the model parameters and reading skill.


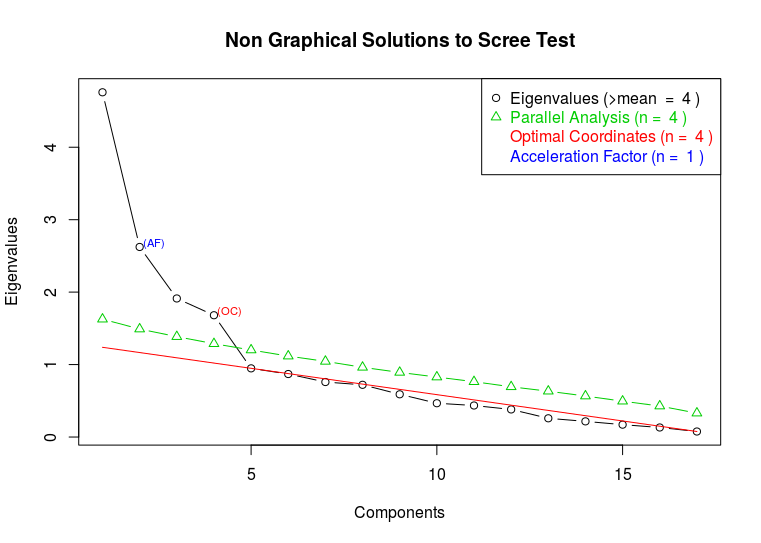


**Figure S5.** *Scree test for exploratory factor analysis. Four standard measures of model fit are given: eigenvalues, parallel analysis, the optimal coordinates metric and acceleration factor metric.*


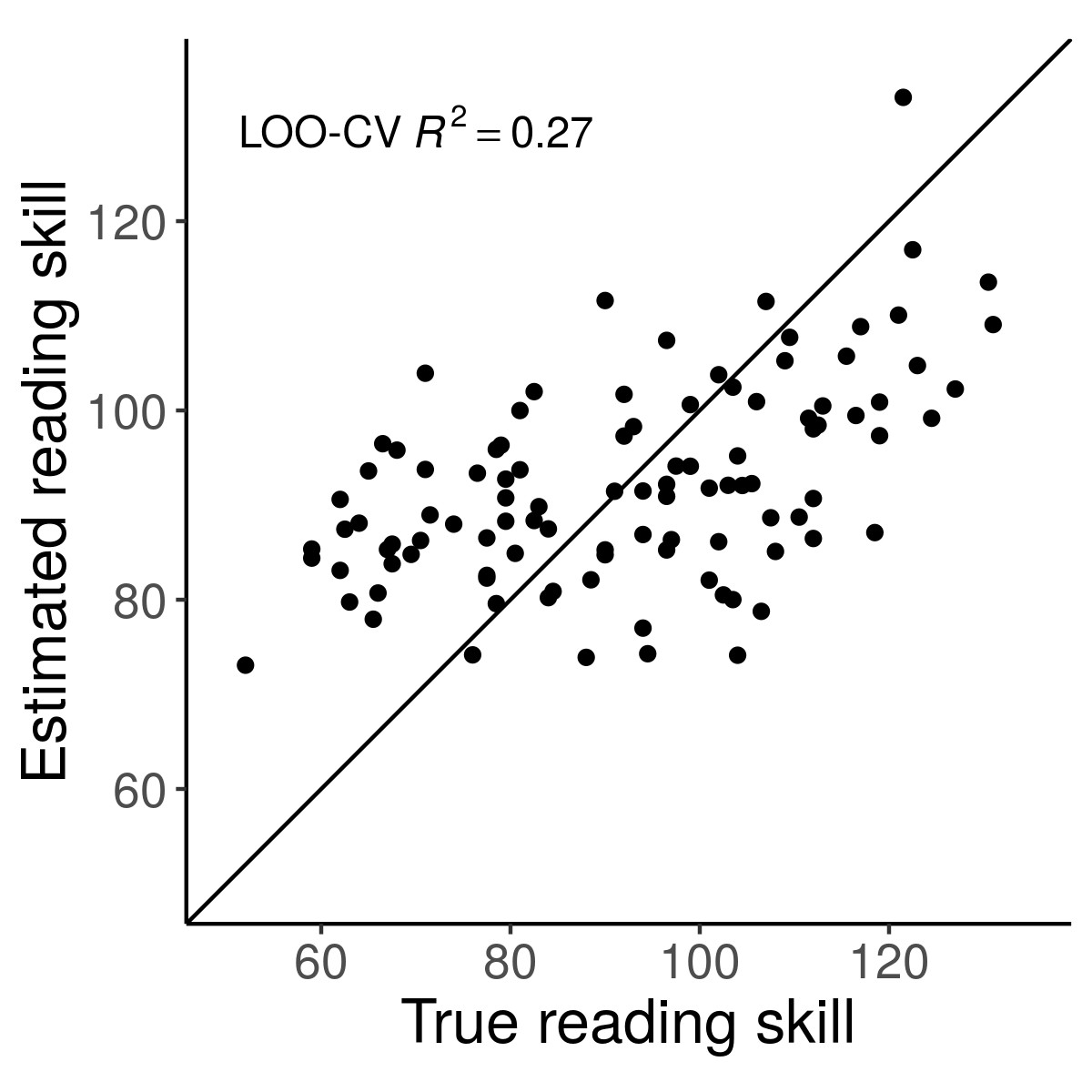


**Figure S6.** *Comparison of true versus predicted reading skill for the single-factor model. Point estimates are computed with leave-one-out cross validation (LOO-CV).*
